# Variation in histone configurations correlates with gene expression across nine inbred strains of mice

**DOI:** 10.1101/gr.277467.122

**Published:** 2023-06

**Authors:** Anna L. Tyler, Catrina Spruce, Romy Kursawe, Annat Haber, Robyn L. Ball, Wendy A. Pitman, Alexander D. Fine, Narayanan Raghupathy, Michael Walker, Vivek M. Philip, Christopher L. Baker, J. Matthew Mahoney, Gary A. Churchill, Jennifer J. Trowbridge, Michael L. Stitzel, Kenneth Paigen, Petko M. Petkov, Gregory W. Carter

**Affiliations:** 1 The Jackson Laboratory for Mammalian Genetics, Bar Harbor, Maine 04609, USA;; 2 The Jackson Laboratory for Genomic Medicine, Farmington, Connecticut 06032, USA

## Abstract

The Diversity Outbred (DO) mice and their inbred founders are widely used models of human disease. However, although the genetic diversity of these mice has been well documented, their epigenetic diversity has not. Epigenetic modifications, such as histone modifications and DNA methylation, are important regulators of gene expression and, as such, are a critical mechanistic link between genotype and phenotype. Therefore, creating a map of epigenetic modifications in the DO mice and their founders is an important step toward understanding mechanisms of gene regulation and the link to disease in this widely used resource. To this end, we performed a strain survey of epigenetic modifications in hepatocytes of the DO founders. We surveyed four histone modifications (H3K4me1, H3K4me3, H3K27me3, and H3K27ac), as well as DNA methylation. We used ChromHMM to identify 14 chromatin states, each of which represents a distinct combination of the four histone modifications. We found that the epigenetic landscape is highly variable across the DO founders and is associated with variation in gene expression across strains. We found that epigenetic state imputed into a population of DO mice recapitulated the association with gene expression seen in the founders, suggesting that both histone modifications and DNA methylation are highly heritable mechanisms of gene expression regulation. We illustrate how DO gene expression can be aligned with inbred epigenetic states to identify putative *cis*-regulatory regions. Finally, we provide a data resource that documents strain-specific variation in the chromatin state and DNA methylation in hepatocytes across nine widely used strains of laboratory mice.

The development of the Diversity Outbred (DO) mice (Churchill et al. 2012; Svenson et al. 2012; Kebede and Attie 2014; Bogue et al. 2015; Keller et al. 2019; Kurtz et al. 2020; Koyuncu et al. 2021) and their sister population, the Collaborative Cross (CC) (Durrant et al. 2011; Threadgill et al. 2011; Threadgill and Churchill 2012; Mao et al. 2015; Graham et al. 2021), has shown the critical importance of genetic diversity in our understanding of disease biology. These mice have been used to investigate the genetic architecture of complex disease (Tyler et al. 2017), to identify genetic modifiers of Mendelian disease (Takemon et al. 2021), and to study the effects of genetic variation on susceptibility to infectious disease (Kurtz et al. 2020). These models have the potential to uncover mechanistic insights into multiple aspects of human health and disease. However, although the genetic diversity of these mice is well documented, the epigenetic diversity of these strains is relatively unknown.

Epigenetic modifications, such as histone modifications (Godini et al. 2018; Xu et al. 2021) and DNA methylation (Ji et al. 2010; Wiench et al. 2011), regulate gene expression by modifying the accessibility of DNA to transcription machinery (Jones 2012; Moore et al. 2013; Lawrence et al. 2016). These modifications vary across cell types, allowing organisms to develop all of their diverse cells from a single genome. Epigenetic modifications have also been shown to vary across individuals in humans (McVicker et al. 2013; Kang et al. 2021), rats (Rintisch et al. 2014), cattle (Prowse-Wilkins et al. 2022), and mice, including some of the DO/CC founders (Schilling et al. 2009; Xie et al. 2012; Gujar et al. 2018; Link et al. 2018; Grimm et al. 2019; Zhou et al. 2022). This epigenetic variation across individuals has been shown to be heritable (Schilling et al. 2009; Grimm et al. 2019) and to be associated with variation in gene expression (Rintisch et al. 2014; Kang et al. 2021; Prowse-Wilkins et al. 2022), cellular phenotypes (Link et al. 2018), and clinical outcomes (Kang et al. 2021; Hawe et al. 2022).

Regulation of gene expression through heritable epigenetic variation is thus an important link between genotype and phenotype. Because the majority of disease-associated genetic variants discovered in humans are in gene regulatory regions, it has been suggested that it is the regulation of gene expression, rather than alteration of protein function, that is the primary mechanism through which genetic variants confer disease risk (Hindorff et al. 2009; Pennisi 2011; Maurano et al. 2012; Farh et al. 2015). Therefore, having well-annotated maps of epigenetic modifications in disease models like the DO/CC founders is potentially critical to understanding mechanisms of gene regulation and its impact on disease.

To extend documented epigenetic variation to all DO/CC founders, we undertook a strain survey of epigenetic variation in hepatocytes across the eight founders of the DO/CC mice, as well as DBA/2J, which, along with C57BL/6J, is one of the founders of the widely used BXD recombinant inbred panel of mice (Ashbrook et al. 2021).

We assayed four histone modifications: H3K4me3, which is associated with promoter regions (Bernstein et al. 2005; Heintzman et al. 2007); H3K4me1, which is associated with enhancer regions (Heintzman et al. 2007); H3K27me3, which is associated with polycomb repression (Bonasio et al. 2010); and H3K27ac, which has been associated with active enhancers and promoters (Heintzman et al. 2009; Creyghton et al. 2010; Rada-Iglesias et al. 2011). We also assayed DNA methylation, which is differentially associated with gene expression depending on its position relative to the gene (Jones 2012; Moore et al. 2013). Methylation of DNA in promoters inactivates the promoters, thereby reducing gene expression, whereas methylation of DNA in insulators inactivates the insulators, thereby increasing expression of the targeted gene (Jones 2012).

We used ChromHMM (Ernst and Kellis 2012) to identify 14 chromatin states, each representing a unique combination of the four histone marks. We investigated the association between variation in these epigenetic markers and variation in gene expression across the nine inbred strains.

We extended our analysis into a population of DO mice (Churchill et al. 2012; Svenson et al. 2012; Gatti et al. 2014; Chick et al. 2016) to investigate the heritability of histone modifications and DNA methylation with respect to gene expression. To do this, we imputed the 14 chromatin states and DNA methylation into the DO mice. We then mapped gene expression to the imputed epigenetic states to assess the extent to which gene expression in the DO mice corresponded with imputed epigenetic variation.

## Results

**Figure 1.**
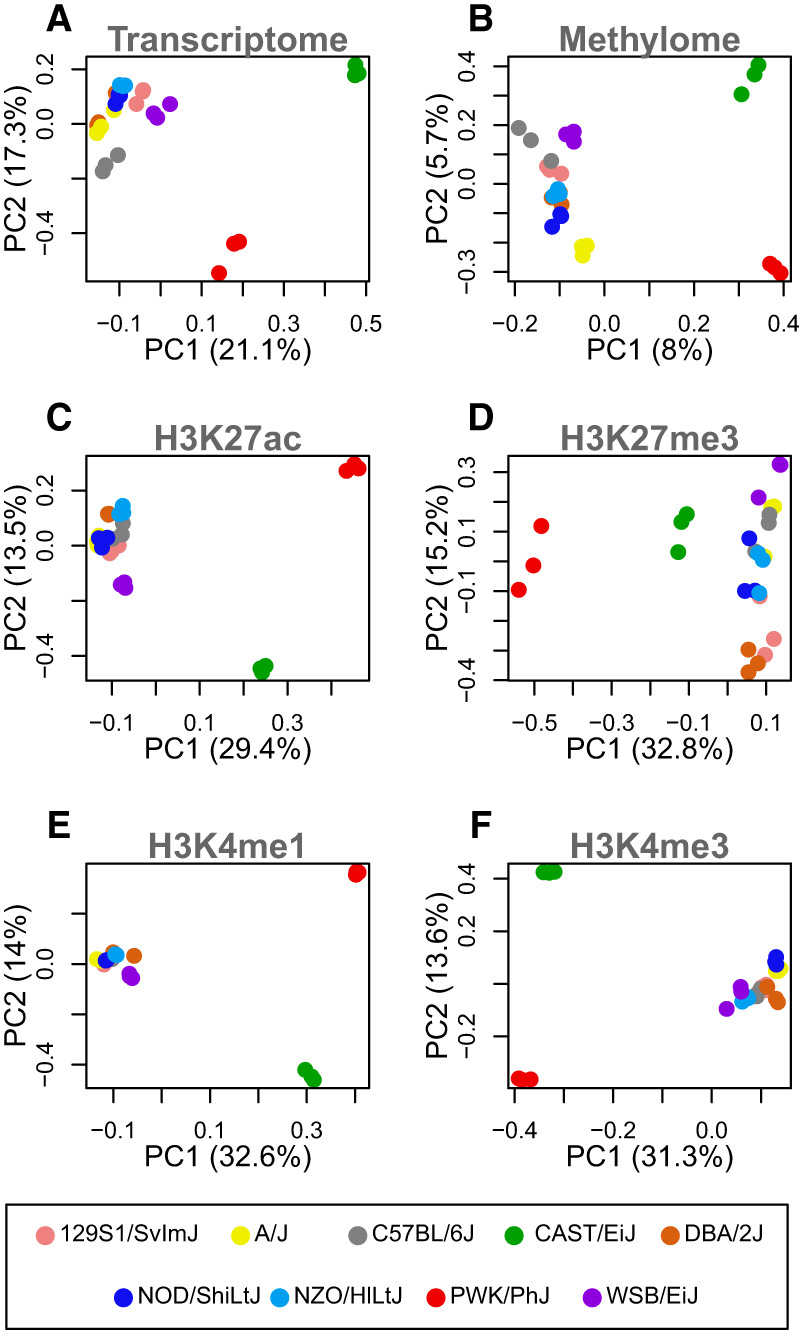
The first two principal components of each genomic feature across nine inbred mouse strains. In all panels, each point represents an individual mouse, and strain is indicated by color as shown in the legend at the *bottom* of the figure. Three individuals per strain are shown. Each panel is labeled with the data used to generate the PC plot. (*A*) Hepatocyte transcriptome: all transcripts expressed in isolated hepatocytes. (*B*) DNA methylation: the percentage of methylation at all CpG sites shared across all individuals. (*C*–*F*) Histone modifications: the peak heights of the indicated histone modification for positions aligned across strains.

Both gene expression and epigenetic state were consistent within each inbred mouse strain but varied across the strains, suggesting strong genetic regulation of both modalities. This is seen as a clustering of individuals from the same strain in principal component plots of transcriptomic and epigenetic features (Fig. 1). Patterns of gene expression (Fig. 1A), DNA methylation (Fig. 1B), and individual histone modifications (Fig. 1C–F) clustered in similar patterns, although a relatively small percentage of the variation in the methylome was related to strain. The three subspecies *musculus* (in red), *castaneous* (in green), and *domesticus* (all others) were widely separated, suggesting that subspecies structure made up the majority of the observed variance. The *domesticus* strains largely clustered together. These data provide evidence that epigenetic features relate to gene expression in a manner that is consistent with the subspecific origin of the mouse strains (Yang et al. 2007). For a more detailed visualization of the correlations between strains, see Supplemental Figure S1. Also, note that all genes used in this analysis were expressed at a minimal level across the strains (overall mean of 5 TPM), so results do not include data from nonexpressed genes.

### Chromatin state overview

**Figure 2.**
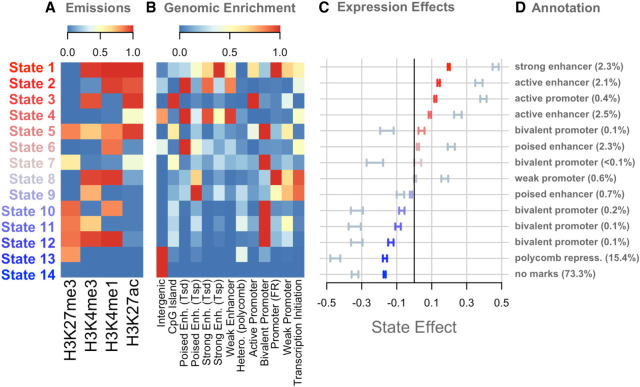
Overview of chromatin state composition, genomic distribution, and association with expression. (*A*) Emission probabilities for each histone modification in each chromatin state. Blue indicates the absence of the histone modification, and red indicates the presence of the modification. (*B*) The distribution of each state around functional elements in the genome. Red indicates that the state is enriched near the annotated functional element. Blue indicates that the state is depleted near the annotated functional element. Rows were scaled to run between zero and one for ease of visualization. Abbreviations are as follows: (Enh.) Enhancer, (Tsd) distal to the transcription start site, (Tsp) proximal to the transcription start site, (Hetero.) heterochromatin, (FR) flanking region. (*C*) The association between chromatin state variation and gene expression. Bars are colored based on the size and direction of the state's association with expression. Red/blue bars show the associations of chromatin state with gene expression across strains. Blue-gray bars show the associations of chromatin state with gene expression across genes. (*D*) Plausible annotations for each state based on genomic enrichments and association with gene expression. The numbers in parentheses indicate the percentage of the genome that was assigned to each state. (repress.) Repressor.

We used ChromHMM to identify 14 chromatin states composed of unique combinations of the four histone modifications (Fig. 2A). We calculated the enrichment of each state near predicted functional elements in the mouse liver (Fig. 2B; Supplemental Fig. S2) and correlated the presence of each state with gene expression both across genes and across the inbred strains (Fig. 2C).

To associate chromatin state with expression across transcripts (Fig. 2C, blue-gray bars), we calculated the proportion of each gene body that was occupied by each state in each inbred strain. We then fit a linear model to associate the proportion of each chromatin state with the amount of transcription (Methods). We did this separately in each strain. Some chromatin states, such as state 1, were more abundant in highly expressed genes, whereas other states, such as state 13, were more abundant in lowly expressed genes.

We compared this correlation to the correlation between chromatin state and gene expression across strains (Methods) (Fig. 2C, red/blue bars). To do this, we normalized the expression of each transcript and the proportion of each chromatin state across strains (Methods). We then fit a linear model to estimate whether the proportion of each state varying across strains was associated with gene expression. For any given transcript, strains with greater proportions of state 1 had higher expression than strains with lower proportions of state 1. Through this calculation, we can associate strain variation in chromatin state with strain variation in gene expression.

In Figure 2, the states are ordered by their association with gene expression across strains, which helps illustrate several patterns. Overall, states that were associated with increased expression across transcripts were also associated with increased expression when varying across strains. The state with the largest negative association with gene expression across strains, state 14, was characterized by the absence of all measured modifications. Other states associated with reduced gene expression contained the repressive mark H3K27me3. The states with the largest positive correlations with gene expression all had some combination of the activating marks H3K4me3, H3K4me1, and H3K27ac. The repressive mark was less commonly seen in these activating states.

We used the functional element enrichments to assign putative annotations to each of the 14 chromatin states (Fig. 2D). Except for state 14, all states were enriched around at least one of the predicted functional elements in the mouse liver (Fig. 2B). Where there was more than one obvious enrichment for the state, we used our own associations with gene expression to narrow down which regulatory label we assigned each state. The enrichments of these states largely matched the associations we saw between each state and gene expression (Fig. 2C). For example, state 1, which was enriched around strong enhancers, was the state that was most strongly correlated with increased expression both across genes, as well as across strains. Likewise, states 2–4 were all enriched around active enhancers or promoters and were all correlated with increased expression overall.

At the other end of the spectrum, state 13 was enriched around polycomb repressor marks, as we would expect because it was defined by presence of H3K27me3, which is associated with polycomb repression. This state was also correlated with reduced expression both across genes and across strains.

Many of the states with weaker associations with gene expression, both positive and negative, were most enriched around bivalent promoters. This suggests that the bivalent promoter class may represent a diverse array of functional elements with varied effects on gene expression and that more detailed experiments investigating the relationship between these states and gene expression could potentially identify novel chromatin states influencing expression in these cells.

### DNA methylation overview

To investigate the variation in DNA methylation across the inbred strains, we examined both strain-specific CpG sites and strain-specific methylation values. We defined a strain-specific CpG site as one that was present in all individuals in at least one strain and absent in all individuals in at least one other strain.

**Figure 3.**
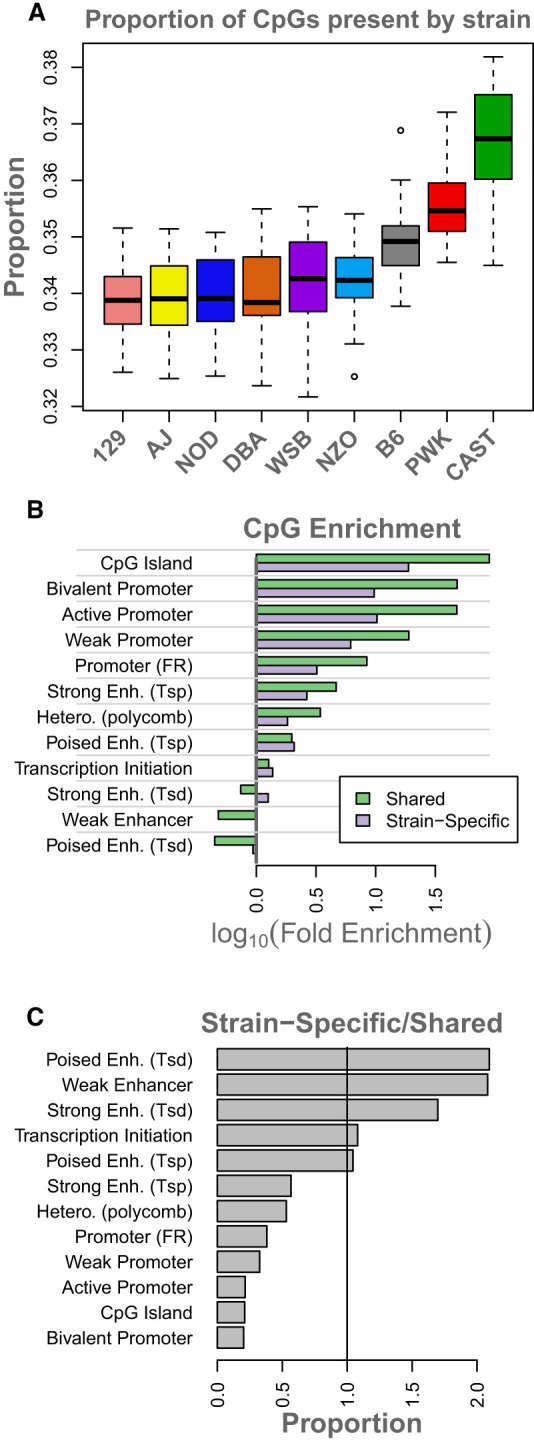
Overview of strain-specific CpG sites. (*A*) Boxes show the proportion of strain-specific CpG sites that is present in each strain. Boxes are colored by official strain colors for ease of visualization. Short names for strains are indicated *below* each box. (*B*) The *log*_10_ (fold enrichment) of CpG sites shared across all strains (green) and those that are strain specific (purple). (*C*) A comparison of enrichments between CpG sites that are shared across all strains and those that are strain specific. Bars above one show where strain-specific CpGs were more enriched than shared CpGs. Bars below one indicate where strain-specific CpGs were less enriched than shared CpGs. The vertical line marks where shared and strain-specific CpGs were equally enriched. Abbreviations are as follows: (FR) flanking region, (Tsp) transcription start site proximal, (Tsd) transcription start site distal, (Hetero.) heterochromatin, (Enh.) enhancer.

Roughly 17.8% of all CpG sites were strain specific, ranging from 16% to 19% across the chromosomes. Strain-specific CpG sites were more commonly present in CAST, PWK, and B6 compared with the other strains (Fig. 3A).

CpG sites that were shared across all strains were enriched around genomic features such as CpG islands and promoters (Methods) (Fig. 3B, green). Strain-specific CpG sites were also enriched around CpG islands and promoters (Fig. 3B, purple). However, relative to the CpG sites found in all strains, the strain-specific CpG sites were more strongly enriched specifically in enhancers, especially transcription start site (TSS)-distal poised enhancers and weak enhancers (Fig. 3C). Relative to the CpG sites common across all strains, strain-specific sites were depleted in promoter regions and CpG islands (Fig. 3C), suggesting that variation in DNA methylation across strains primarily occurs in enhancers that fine-tune gene expression levels rather than in promoters, which might result in genes being turned on or off.

### Spatial distribution of epigenetic modifications around gene bodies

**Figure 4.**
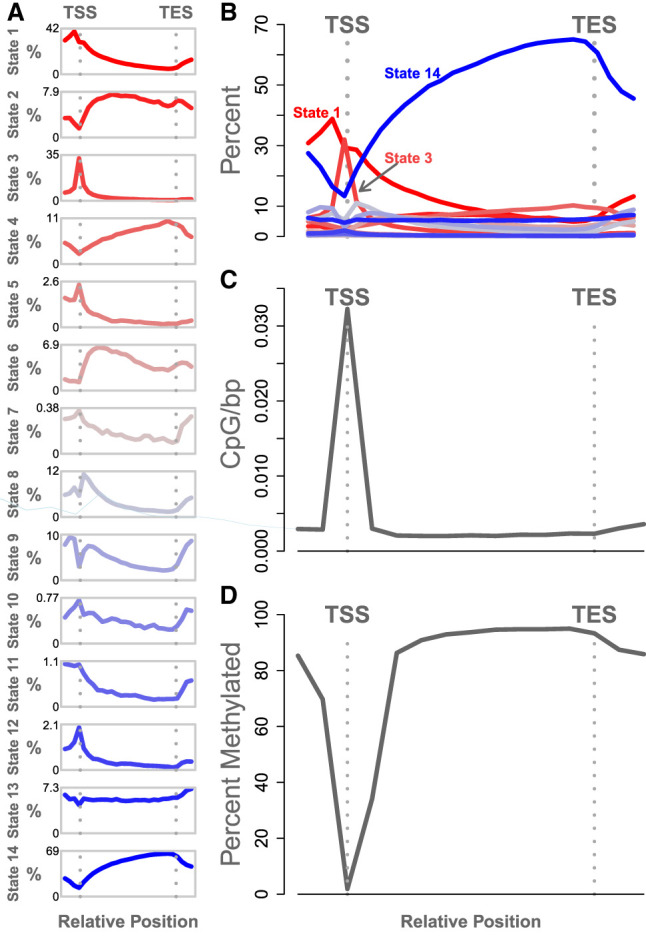
Relative abundance of chromatin states and methylated DNA. (*A*) Each panel shows the abundance of a single chromatin state relative to gene transcription start site (TSS) and transcription end site (TES). The *y*-axis in each panel is the percentage of genes containing the state. Each panel has an independent *y*-axis to better show the shape of each curve. The *x*-axis is the relative gene position. The TSS and TES are marked as vertical gray dotted lines. (*B*) The same data shown in panel *A*, but with all states overlaid onto a single set of axes to show the relative abundance of the states. (*C*) The density of CpG sites relative to the gene body. The *y*-axis shows the inverse inter-CpG distance in base pairs. The density is highest near the TSS. CpG sites are less dense within the gene body and in the intergenic space. (*D*) Percentage of methylation relative to the gene body. The *y*-axis shows the median percentage of methylation at CpG sites, and the *x*-axis shows relative gene position. CpG sites near the TSS are unmethylated relative to intragenic sites and to sites just upstream of and downstream from the gene bodies. In both *C* and *D*, standard error is shown as a blue envelope around the mean; however, the standard error is so small that it is not visible in the figure.

In addition to looking for enrichment of chromatin states and CpG sites near annotated functional elements, we characterized the fine-grained spatial distribution of these features around gene bodies by normalizing genomic positions to run from zero at the TSS to one at the transcription end site (TES; see Methods) (Fig. 4).

The spatial patterns of the individual chromatin states are shown in Figure 4A, and an overlay of all states together (Fig. 4B) emphasizes the difference in abundance between the most abundant states (states 1, 3, and 14) and the remaining states, which were relatively rare.

Each chromatin state had a characteristic distribution pattern across the gene body. For example, state 14, which was characterized by the absence of all measured histone modifications, was strongly depleted near the TSS, indicating that this region is commonly subject to the histone modifications we measured here. It should be noted that this pattern is independent of the global enrichment patterns shown in Figure 2. Although state 14 is generally depleted in gene bodies relative to intergenic regions, it is especially depleted at the TSS. In contrast, states 1 and 3 were both relatively abundant at the TSS. State 3 was very narrowly concentrated right at the TSS, consistent with its annotation as an active promoter (Fig. 2). State 1, on the other hand, was especially enriched just upstream of the TSS, consistent with its annotation of a TSS-proximal strong enhancer. State 2 was depleted near the TSS but enriched within the gene body, consistent with its annotation of a TSS-distal enhancer.

States with weaker associations to expression (indicated by grayer shades in Fig. 4) were of lower abundance but had distinct distribution patterns around the gene body, suggesting the possibility of distinct functional roles in the regulation of gene expression. These abundance patterns were not different across the strains (Supplemental Fig. S3).

DNA methylation showed similar characteristic variation in abundance (Fig. 4C,D). The TSS had densely packed CpG sites relative to the gene body (Fig. 4C). As expected, the median CpG site near the TSS was consistently hypomethylated relative to the median CpG site (Fig. 4D). All genes used in this analysis were expressed and thus had some degree of hypomethylation. There were also no large-scale differences in CpG distribution or percentage of methylation across strains (Supplemental Fig. S4).

### Spatially resolved associations with gene expression

**Figure 5.**
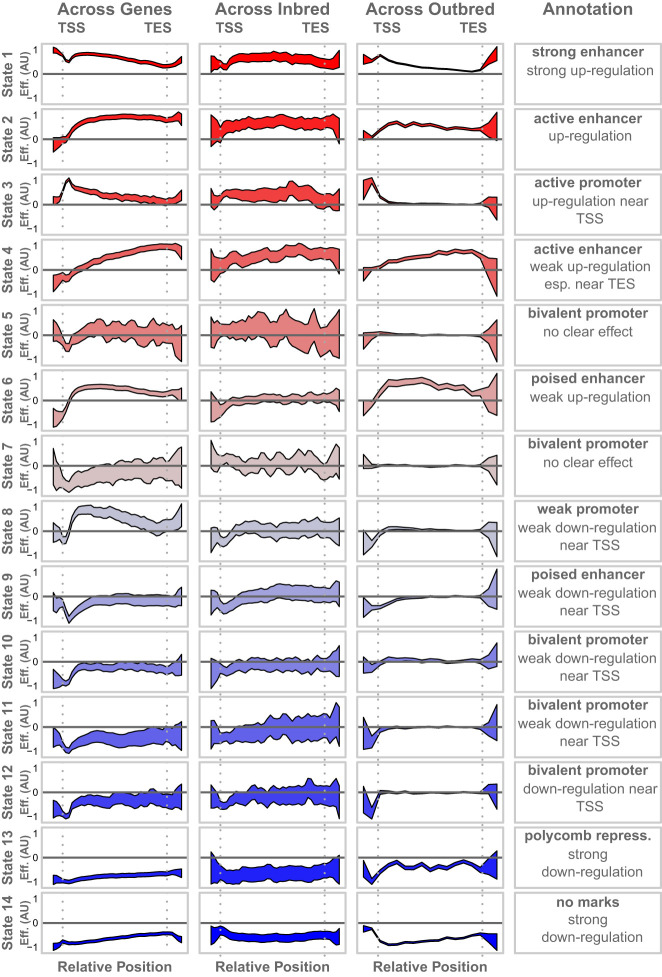
Associations of chromatin states with gene expression. Each column shows the association of each chromatin state with gene expression in a different experimental context as labeled. Effects shown are β coefficients from Equation 1. The *y*-axes vary across each row to emphasize the shape of each effect, so *y*-axis labels indicate only positive and negative effects. Colored areas show the 95% confidence interval around each estimate. The *final* column shows the annotation of each state for comparison with its association with gene expression. All *x*-axes show the relative position along the gene body, running from just upstream of the TSS to just downstream from the TES. Vertical gray dotted lines mark the TSS and TES in all panels.

The distinct spatial distributions of the chromatin states and methylated CpG sites around the gene body raised the question as to whether the associations of these states with gene expression could also be spatially resolved. To investigate this possibility, we tested the association between gene expression and both chromatin state and DNA methylation using spatially resolved models (Methods). We tested the association of each chromatin state with expression across genes within hepatocytes (Fig. 5, left column) and the association of each chromatin state with the variation in gene expression across strains (Fig. 5, middle column).

All chromatin states showed spatially dependent associations with gene expression within hepatocytes. Figure 5 shows how these associations are distributed across the states and across the gene bodies. For many of the states, the associations with expression were concentrated at or near the TSS, whereas in the other states, associations were seen across the whole gene. The direction of the coefficients matched the overall associations of each state seen previously (Fig. 2), but here, we see the effects in finer resolution. For example, state 3 was positively correlated overall with gene expression (Fig. 2C), but in Figure 5, we see that this positive correlation is primarily limited to the region near the TSS, consistent with its annotation as a promoter state.

Further, the spatial associations observed across genes (Fig. 5, left column) were largely recapitulated in the measurements across strains (Fig. 5, middle column). That is, chromatin states that either enhanced or suppressed gene expression across hepatocyte genes were similarly related to variation in expression across strains. This suggests that the genetic differences between strains modify chromatin activity in a manner similar to that used across genes. One notable exception was state 6, whose presence up-regulated genes within hepatocytes but was not associated with expression variation across strains.

**Figure 6.**
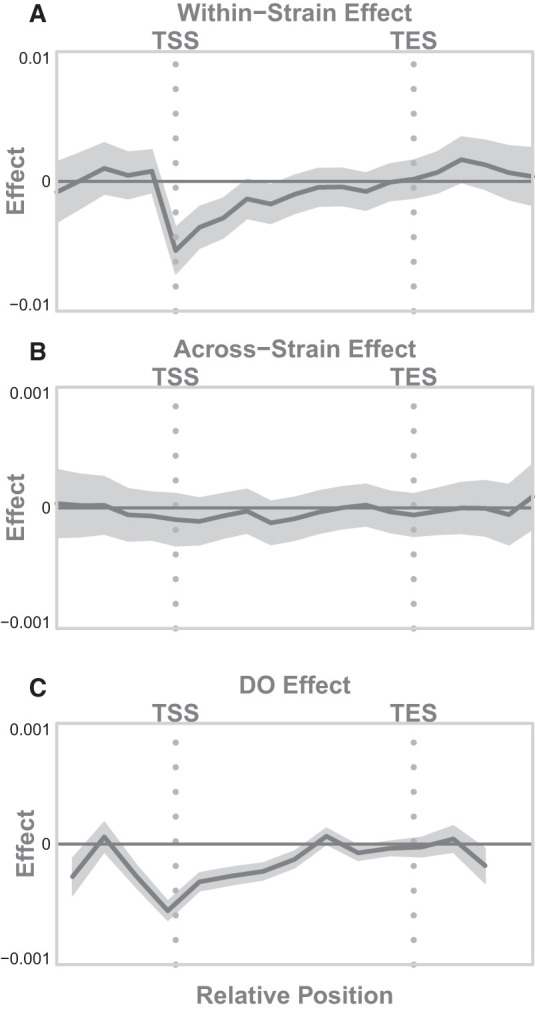
Association of DNA methylation with gene expression (*A*) across genes in hepatocytes, (*B*) across inbred strains, and (*C*) in the DO population. The dark gray line shows the estimated effect of percentage of DNA methylation on gene expression. The *x*-axis is normalized position along the gene body running from the TSS to the TES, marked with vertical gray dotted lines. The horizontal solid black line indicates an association of zero. The shaded gray area shows a 95% confidence interval around the model fit.

We also examined the association of percentage of DNA methylation with gene expression across genes and across strains (Fig. 6). As expected, methylation at the TSS was associated with lower expressed genes in hepatocytes (Fig. 6A). We did not detect an association between DNA methylation percentage and gene expression across inbred strains, perhaps because there were too few strains to reliably estimate the coefficients (Fig. 6B).

### Interactions between chromatin state and DNA methylation

We investigated whether there was an interaction between DNA methylation and chromatin state by asking two questions. First, were CpG sites within different chromatin states methylated at different levels? Second, was DNA methylation within specific chromatin states differentially associated with gene expression across inbred mice? If DNA methylation essentially inactivates a region of DNA, methylation in a region identified as a repressor based on its chromatin state might be expected to increase gene expression, whereas methylation in an active enhancer might decrease gene expression.

To investigate these questions, we identified CpG sites within each of the 14 chromatin states. We calculated the average percentage of methylation of these sites and the association of DNA methylation with gene expression for each set of sites (Methods). We treated missing CpG sites in individual strains as unmethylated.

Although methylation patterns in all states followed roughly the same pattern of being unmethylated at the TSS and methylated within the gene body, values ranged widely across the states from state 3, with a mean of 27% methylated DNA intragenically, to state 14, with 83% methylated DNA intragenically. Again, these differential levels of methylation within these states are consistent with the state annotations. State 3 was annotated as an active promoter, and we would expect DNA methylation in this state to be low. State 14 has no histone modifications and is not expected to be transcriptionally active, which is consistent with high levels of DNA methylation.

DNA methylation within each chromatin state was differentially correlated with gene expression (Supplemental Fig. S5). DNA methylation in state 3, the active promoter state, was associated with decreased gene expression, suggesting that DNA methylation in this state deactivated the active promoter state. Overall, the repressor state, state 13, was negatively associated with gene expression. However, DNA methylation in this state was positively associated with gene expression, suggesting that this repressive state can be inactivated by DNA methylation.

### Imputed chromatin state is associated with gene expression in DO mice

Thus far, we have shown correlations between gene expression and epigenetic features in inbred mice. We were also interested in whether chromatin state and DNA methylation were associated with gene expression in an outbred mouse population. Although we did not measure epigenetic modifications directly in an outbred population, we had liver gene expression from a previously published population of DO mice (Tyler et al. 2017). Inheritance of chromatin state and DNA methylation is complex (Rintisch et al. 2014); however, there is evidence that the heritability for both epigenetic features is high (Fraga et al. 2005; Villicaña and Bell 2021), suggesting the possibility of imputing epigenetic features from local genotype into the DO mice. Even with imperfect estimates of epigenetic features in the outbred mice, a common pattern of association between outbred and inbred mice would support the idea that inherited variance in epigenetic features contributes to inherited variation in gene expression across genetically distinct individuals.

We imputed chromatin state, DNA methylation, and SNPs into the DO population (Methods). Because any feature imputed from a haplotype will be correlated with anything that haplotype is correlated with, we performed permutations that shuffled the relationship between haplotype and chromatin state (Methods). The resulting *P*-value distributions of each genomic feature suggested that each imputed feature was significantly associated with gene expression in the DO beyond the effects of the imputation alone (Supplemental Fig. S6).

We then tested the association between each imputed chromatin state, SNP, or CpG site with gene expression in the DO. We tested each chromatin state independently. The standard method for testing associations is to include independent variables for all alleles (or chromatin states) in a single linear model. However, because there are varying numbers of predictor states across modalities (eight haplotypes, 14 chromatin states, three DNA methylation values, and two SNPs), variance explained across the modalities is not comparable unless the degrees of freedom are equal for all tests. Thus, for all features, we tested only a single haplotype, chromatin state, etc., versus all other possibilities in each model.

**Figure 7.**
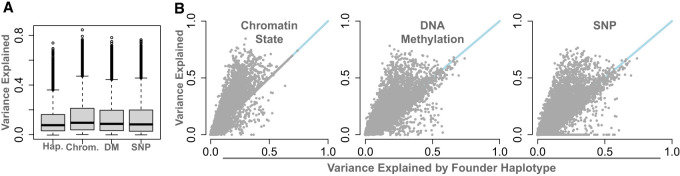
Comparison of the variance explained in DO gene expression by four genomic features: haplotype (Hap.), chromatin state (Chrom.), local SNP genotype (SNP), and local imputed DNA methylation status (DM). (*A*) Distributions of gene expression variance explained by each feature. (*B*) Direct comparisons of variance explained by local haplotype and each of the other genomic features. Blue lines show *y* = *x*. Each point is a single transcript.

Figure 7 compares the variance explained by individual haplotypes with that explained by any individual chromatin state, CpG site, or SNP. All imputed features—individual chromatin states (mean, 14%), DNA methylation (mean, 14%), and SNPs (mean, 13%)—explained more variance in gene expression than individual haplotypes (mean, 11%) (Fig. 7A). This suggests that any given chromatin state, CpG site, or SNP carries more functional information than any individual haplotype, which is primarily a measurement of ancestry.

Figure 7B shows the maximum variance explained by each genomic feature for each transcript in the transcriptome. Dots above the line indicate transcripts for which the imputed genomic feature explained more variance than haplotype. Dots below the line indicate transcripts for which the imputed genomic feature explained less variance than the haplotype. The individual haplotype explained less variance than any other genomic feature for the majority of transcripts. supporting the hypothesis that all these features carry heritable information that potentially regulates gene expression in this genetically diverse population.

To maximize power to estimate associations between epigenetic states and gene expression, we used all animals in the DO population and regressed out the effects of sex and diet from all variables before testing for associations. However, because the inbred animals used in this study were females maintained on a chow diet, it is possible that variation in either sex or diet in the DO population could affect the results. To test whether sex or diet had any effect on the associations between epigenetic features and gene expression, we performed all tests using only females and, again, only with chow-fed animals. Results were similar across these subsets, and any differences in means were within a fraction of a standard deviation of the distributions (Supplemental Fig. S7).

In addition to calculating overall associations, we calculated position-based associations between each epigenetic feature and gene expression (Fig. 5, right column; Fig. 6C). The associations in the DO mice largely matched those seen in the inbred mice for both chromatin state and DNA methylation. Even though DNA methylation showed no association with gene expression across strains in the inbred mice, there was a weak, but significant, association with gene expression in the DO mice. This may be because of the increased power to detect effects in the 378 DO mice relative to the nine inbred strains.

### Hypothesis generation for *cis*-regulatory regions

**Figure 8.**
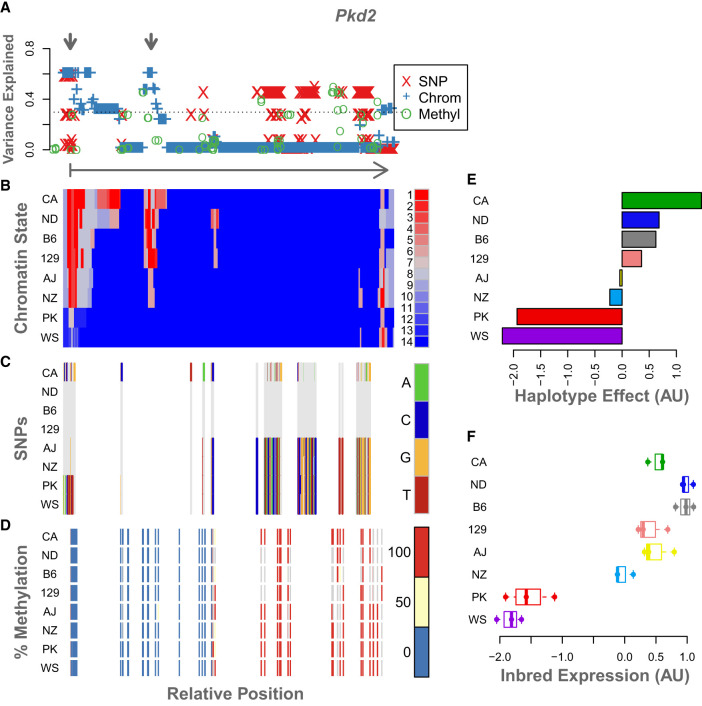
Example of epigenetic states and imputation results for a single gene, *Pkd2*. The legend for each panel is displayed to its *right*. (*A*) The variance in DO gene expression explained at each position along the gene body by each of the imputed genomic features: SNPs, red X's; chromatin state, blue plus signs; and percentage of methylation, green circles. The horizontal dotted line shows the maximum variance explained by any individual haplotype (in this case, CAST). For reference, the arrow *below* this panel runs from the TSS of *Pkd2* (vertical bar) to the TES (arrowhead) and shows the direction of transcription. The gray arrows at the *top* indicate two regions of interest where chromatin state explains height amounts of variance in gene expression. (*B*) The chromatin states assigned to each 200-bp window in this gene for each inbred mouse strain. States are colored by their association with gene expression in the inbred mice. Red indicates a positive association with gene expression, and blue indicates a negative association. Each row shows the chromatin states for a single inbred strain, which is indicated by the label on the *left*. (*C*) SNPs along the gene body for each inbred strain. The reference genotype is shown in gray. SNPs are colored by genotype as shown in the legend. (*D*) Percentage of DNA methylation for each inbred strain along the *Pkd2* gene body. Percentages are binned into 0% (blue), 50% (yellow), and 100% (red). (*E*) Association of haplotype with expression of *Pkd2* in the DO. Haplotype effects are colored by the allele from which they were derived. (*F*) *Pkd2* expression levels across inbred mouse strains. For ease of comparison, panels *B* through *F* are shown in the same order as the haplotype effects.

By aligning associations with gene expression from the DO mice with inbred epigenetic features, we can generate hypotheses about heritable *cis*-regulatory regions in these mice. In particular, for any gene whose variance was explained at least as well by an imputed feature as by haplotype, there is the possibility that the imputed feature marks a *cis*-regulatory element. This occurrence provides an opportunity to annotate novel functional elements in the mouse genome or provide supportive evidence of previously predicted functional elements. As an example, we investigated the gene *Pkd2* (Fig. 8). This gene had a strong local eQTL (LOD = 144.8) that had been previously identified (Chick et al. 2016; Gatti et al. 2017), and large amounts of variance explained (R^2^ = 0.6) by both chromatin state and SNPs (Fig. 8A).

WSB and PWK were low-expressing strains for *Pkd2*, and the remaining strains had higher expression (Fig. 8F). The haplotype effects in the DO mirror this pattern, with the CAST allele showing an especially high association with increased gene expression (Fig. 8E). Figure 8, B through D, shows chromatin state, SNP genotype, and DNA methylation state along the body of *Pkd2*, respectively. Panel A shows the association of each of the imputed features with gene expression in the DO. The detailed view of this gene identified two regions marked by gray arrows in panel A. One is at the TSS and the immediately surrounding area, and the other is just downstream from the TSS.

Both chromatin state and SNPs in these two regions were strongly associated DO expression levels of *Pkd2* (Fig. 8A). Comparing these regions marked in panel A to the chromatin states in panel B, we see that these two regions both have activating chromatin states in the high-expressing haplotypes and an absence of activating marks in the low-expressing haplotypes. We therefore hypothesized that these two regions are heritable *cis*-regulatory regions for *Pkd2*.

The spatial patterns in the SNPs (Fig. 8C) partially mirror those in chromatin state (Fig. 8B). SNPs underlying the more proximal enhancer region could potentially influence gene expression by altering local chromatin state. However, the more distal putative *cis*-regulatory region has no underlying SNPs, suggesting that there is an alternative mechanism for determining chromatin state at this location. Perhaps SNPs in the TSS region regulate chromatin state in both regions. For this particular gene, variation in DNA methylation (Fig. 8D) was not associated with *Pkd2* expression in the DO.

## Discussion

In this study, we showed that the epigenetic landscape of hepatocytes varied widely across commonly used inbred mouse strains and that this variation was associated with strain differences in gene expression. We saw evidence that both (1) chromatin state defined by combinatorial histone modifications and (2) DNA methylation were heritable mechanisms contributing to inter-individual variation in gene expression in mice. For DNA methylation, heritable variation was driven in part by strain-specific CpG sites. These CpG sites were enriched in enhancers, specifically, weak, strong, and poised enhancers distal to the TSS. Strain-specific CpG sites were depleted in promoter regions and CpG islands, suggesting that these regions are more highly conserved across the inbred strains studied here and that enhancer regions are the most diverged. This divergence of CpG sites in enhancer regions results in small variations in gene expression across strains relative to potentially large or catastrophic changes that might be expected with loss or gain of CpG sites in promoter regions.

The chromatin states we identified were represented by combinations of histone modifications that were enriched around previously predicted chromatin states in the mouse liver. We used these enrichments to annotate each state but noted that the annotations agreed both with relative abundance around the gene body and with associations to gene expression.

Five of the 14 states we identified were enriched around bivalent promoters. Bivalent states are characterized by a combination of activating and repressing histone modifications (Vastenhouw and Schier 2012; Voigt et al. 2013). Consistent with this definition, all five states included the repressive mark, H3K27me3, and at least one of the activating marks. All of these states were also most abundant around the gene TSS, further supporting the annotation of promoter. Three of these states, states 10, 11, and 12, were associated with reduced gene expression both across genes and across strains, suggesting that these states marked genes that were poised for expression but were not highly expressed. These associations were replicated in the DO for states 11 and 12, suggesting that these states represented a heritable form of gene expression regulation.

Bivalent promoters are typically considered dynamic states that change over the course of differentiation and in response to external stimuli. These regulatory regions have been studied primarily in the context of development. They are abundant in undifferentiated cells and are often resolved either to active promoters or to silenced promoters as the cells differentiate into their final state (Vastenhouw and Schier 2012; Voigt et al. 2013). These promoters have also been shown to be important in the response to changes in the environment: Their abundance increases in breast cancer cells in response to hypoxia (Prickaerts et al. 2016). It is therefore notable to see apparently heritable bivalent promoters in differentiated hepatocytes. Genes marked by state 11 were enriched for mesodermal cell differentiation and Notch signaling, suggesting a developmental role for this state. Similarly, genes marked by state 12 were enriched for blood vessel and endothelial morphogenesis, as well as Wnt signaling.

That we identified these states in differentiated hepatocytes may indicate that a subset of developmental genes retains the ability to be activated under certain circumstances, such as during liver regeneration in response to injury. Both Wnt signaling and Notch signaling are involved in wound repair (Chigurupati et al. 2007; Whyte et al. 2012; Shi et al. 2015) and liver regeneration (Thompson and Monga 2007; Yue et al. 2018; Hu and Monga 2021). The observation that these states likely represent a heritable form of *cis*-regulation is intriguing and may suggest heritable variation in response to liver injury or convergent evolution of regeneration pathways.

State 5 was also annotated as a bivalent promoter, but the evidence for this annotation was less clear than for the other states with this annotation. State 5 was enriched primarily around predicted bivalent promoters in the mouse liver (Fig. 2). However, it also included the presence of H3K27ac, which is typically associated with active enhancers rather than inactive bivalent promoters (Creyghton et al. 2010; Voigt et al. 2013). The association of state 5 with gene expression was also inconsistent. This state was associated with lower gene expression in hepatocytes but with higher gene expression when looking across strains. That is, genes with state 5 were more lowly expressed than other hepatocyte genes, but for any given gene, strains with state 5 had higher expression than strains with other states in the same position.

The association of state 5 with reduced expression within hepatocytes is consistent with the annotation of bivalent promoter. Genes marked with this state were enriched for vascular development and Wnt signaling, further supporting the annotation. When positions marked with state 5 varied across strains, the most common alternate state at these positions was state 12, another bivalent promoter. Thus, this group of genes, in general, was down-regulated relative to other genes. However, our results suggest that state 5 was associated with less severe down-regulation compared with state 12, resulting in an apparent up-regulation when looking across strains. It is also possible that the inconsistent results observed for state 5 indicate that it was a mixture of state 12 and another state. State 5 had a very similar abundance distribution, effect size distribution, and GO term enrichments to those of state 12. As a whole, the group of states annotated as bivalent promoters raises the intriguing possibility of identifying new modes of expression regulation through histone modification. Although these five states all received the same annotation, each had a unique pattern of distribution around the gene body and association with gene expression, suggesting that each represents a different functional element in the mouse genome.

The diversity in the associations with gene expression observed across all 14 chromatin states highlights the importance of analyzing combinatorial states as opposed to individual histone modifications. The three states with the largest positive associations with transcription each had a distinct combination of the three activating histone marks: H3K4me1, H3K4me3, and H3K27ac. And although all three states were associated with increased gene expression, each had a distinct spatial distribution. This variation in spatial distribution was mirrored in the spatial associations with transcription. The distinct patterns among these states would not be detectable without analysis of the histone modifications in combination. These results highlight the complexity of the histone code and the importance of analyzing combinatorial states.

State 9 further illustrates the importance of the combinatorial approach. State 9 was defined as the presence of H3K4me3 and the absence of all other marks. H3K4me3 is most frequently associated with increased transcriptional activity (Santos-Rosa et al. 2002; Schneider et al. 2004; Bernstein et al. 2005; Wysocka et al. 2006), so the association of state 9 with reduced transcription is a deviation from the dominant paradigm. This state was enriched around predicted poised enhancers in the mouse liver data, and genes marked with this state were enriched for functions such as stress response, DNA damage repair, and ncRNA processing. Taken together, these results suggest that this state may be used to regulate subsets of genes involved in responses to environmental stimuli. They further show that the relationship between H3K4me3 and gene expression is more complex than simple activation.

The merging of DO expression quantitative trait loci with inbred chromatin state maps offers a potential method to identify *cis*-regulatory regions. The *Pkd2* example illustrates how this could be performed. Given that there is a *cis*-eQTL at this locus and that imputed chromatin state explained a large amount of variance in DO gene expression, it made sense to look at the patterns of genomic features around this gene. The patterns of chromatin state and SNPs in the gene body pointed to possible molecular mechanisms for the observed eQTL. Both the presence of activating chromatin states and their breadth correlated with gene expression, suggesting the presence of local regulatory regions. The CpG sites in and around these putative regulatory regions are unmethylated across all strains, further supporting the hypothesis that chromatin state in these regions is actively regulating transcription. Validation of these regions is beyond the scope of this study, but our results suggest that combining DO eQTL data with inbred epigenetic data may serve as an important resource in identifying putative regulatory regions.

The discordance between the patterns of chromatin state and SNPs in this gene may also point to potentially novel regulatory mechanisms. Variation in chromatin state at the more distal enhancer is present in the absence of local SNPs. This suggests that the presence of the distal enhancer is determined by another mechanism, perhaps SNPs acting in *trans* to this region, or local variation that was not measured by SNP genotyping, for example, indels. Genetic variation located at a distance from the putative enhancer sites could also potentially alter the 3D configuration of the genome, resulting in variable access of transcription factors to the enhancer.

Broadly, local variation in chromatin state, DNA methylation, and individual SNPs were all more highly correlated with DO gene expression than were individual haplotypes. Individual haplotypes are a measure of ancestry, whereas chromatin state, DNA methylation, and SNPs all potentially functionally related to gene expression. Two haplotypes that are not identical by descent may share a repressor state that is functionally associated with reduced gene expression. These observations raise the possibility of shifting toward mapping traits with functional elements of the genome rather than ancestral allele labels. Many researchers already use SNPs in mapping rather than haplotype, but the set of functional features could be expanded further to include DNA methylation and histone modifications. By combining the power of haplotype mapping with the high-resolution and mechanistic insights of other genomic and epigenomic features, we can begin to build mechanistic hypotheses that link genetic variation to variation in gene expression and physiology.

## Methods

### Ethics statement

All animal procedures followed Association for Assessment and Accreditation of Laboratory Animal Care guidelines and were approved by the institutional animal care and use committee (The Jackson Laboratory, protocol AUS 04008).

### Inbred mice

Three female mice from each of nine inbred strains were used. Eight of these strains (129S1/SvImJ, A/J, C57BL/6J, CAST/EiJ, NOD/ShiLtJ, NZO/HlLtJ, PWK/PhJ, and WSB/EiJ) are the eight strains that served as founders of the CC/DO mice (Chesler et al. 2008). The ninth strain, DBA/2J, will facilitate the interpretation of existing and forthcoming genetic mapping data obtained from the BXD recombinant inbred strain panel. Samples were harvested from the mice at 12 wk of age.

#### Liver perfusion

To purify hepatocytes from the liver cell population, the mouse livers were perfused with 87 CDU/mL Liberase collagenase with 0.02% CaCl_2_ in Leffert's buffer to digest the liver into a single-cell suspension and then isolated using centrifugation.

We aliquoted 5 × 10^6^ cells for each RNA-seq and bisulfite sequencing, and the rest were cross-linked for ChIP assays. Both aliquots were spun down at 200 rpm for 5 min and resuspended in 1200 μL RTL + BME (for RNA-seq) or frozen as a cell pellet in liquid nitrogen (for bisulfite sequencing). In the sample for ChIP-seq, protein complexes were cross-linked to DNA using 37% formaldehyde in methanol. All cell samples were stored at −80°C until used (for more detail, see Supplemental Methods).

#### Hepatocyte histone binding and gene expression assays

Hepatocyte samples were used in the following assays:

1. RNA-seq to quantify mRNA and long noncoding RNA expression, with about 30 million reads per sample.
2. Reduced-representation bisulfate sequencing to identify methylation states of about 2 million CpG sites in the genome. The average read depth was 20–30×.
3. Chromatin immunoprecipitation and sequencing to assess binding of the following histone marks:
  - H3K4me3 to map active promoters,
  - H3K4me1 to identify active and poised enhancers,
  - H3K27me3 to identify polycomb repression,
  - H3K27ac, to identify actively used enhancers, and
  - A negative control (input chromatin).

Samples were sequenced with about 40 million reads per sample.

The samples for RNA-seq in RTL + BME buffer were sent to The Jackson Laboratory Gene Expression Service for RNA extraction and library synthesis.

#### Histone chromatin immunoprecipitation assays

After extraction, hepatocyte cells were lysed to release the nuclei, spun down, and resuspended in 130 µL MNase buffer with 1 mM PMSF (Sigma-Aldrich 78830) and 1× protease inhibitor cocktail (Roche) to prevent histone protein degradation. The samples were then digested with 15 U of micrococcal nuclease (MNase), which digests the exposed DNA but leaves the nucleosome-bound DNA intact. We confirmed digestion of nucleosomes into 150-bp fragments with agarose gel. The digestion reaction was stopped with EDTA, and samples were used immediately in the ChIP assay. The ChIP assay was performed with Dynabead Protein G beads and histone antibodies (H3K4me3, Millipore-Sigma 07-473; H3K4me1, Millipore-Sigma 07-436; H3K27me3, Millipore-Sigma 07-449; H4K27ac, abcam ab4729). After binding to antibodies, samples were washed to remove unbound chromatin and then eluted with high-salt buffer and Proteinase K to digest protein away from DNA–protein complexes. The DNA was purified using the Qiagen PCR purification kit. Quantification was performed using the Qubit quantification system (see Supplemental Methods).

### DO mice

We used previously published data from a population of 478 DO mice (Svenson et al. 2012). DO mice (JAX:DO) are available from The Jackson Laboratory (stock number 009376). The DO population included males and females from DO generations four through 11. Mice were randomly assigned to either a chow diet (6% fat by weight, LabDiet 5K52) or a high-fat, high-sucrose (HF/HS) diet (45% fat, 40% carbohydrates, and 15% protein; Envigo Teklad TD.08811). Mice were maintained on this diet for 26 wk.

#### Genotyping

All DO mice were genotyped as described previously (Svenson et al. 2012) using the Mouse Universal Genotyping Array (MUGA; 7854 markers) and the MegaMUGA (77,642 markers; GeneSeek). All animal procedures were approved by the animal care and use committee at The Jackson Laboratory (animal use summary 06006).

Founder haplotypes were inferred from SNPs using a Hidden Markov Model as described by Gatti et al. (2014). The MUGA and MegaMUGA arrays were merged to create a final set of evenly spaced 64,000 interpolated markers.

#### Tissue collection and gene expression

At euthanasia, whole livers were collected, and gene expression was measured using RNA-seq as described previously (Chick et al. 2016; Tyler et al. 2017). Briefly, hepatocyte RNA was isolated using the TRIzol plus RNA extraction kit (Invitrogen), and 100-bp single-end reads were generated on the Illumina HiSeq 2000.

### Data processing

#### Sequence processing

The raw sequencing data from both RNA-seq and ChIP-seq were put through the quality control program FastQC (0.11.5) (https://www.bioinformatics.babraham.ac.uk/projects/fastqc/), and duplicate sequences were removed before downstream analysis.

#### Transcript quantification

Transcript sequences were aligned to strain-specific pseudogenomes (Chick et al. 2016), which integrate SNPs and indels from each strain based on the GRCm38 mouse genome build. The B6 samples were aligned directly to the reference mouse genome. The pseudogenomes were created using g2gtools (http://churchill-lab.github.io/g2gtools/#overview). We used EMASE (Raghupathy et al. 2018; https://github.com/churchill-lab/emase) to quantify the gene expression counts and DESeq2 vst transformation (Love et al. 2014) to normalize the gene expression data. We filtered out transcripts with <1 CPM in two or more replicates.

#### ChIP-seq quantification

We used MACS 1.4.2 (Zhang et al. 2008) to identify peaks in the ChIP-seq sequencing data, with a significance threshold of *P* ≤ 10^−5^. To compare peaks across strains, we converted the MACS output peak coordinates to common B6 coordinates using g2gtools.

### Quantifying DNA methylation

RRBS data were processed using a Bismark-based pipeline modified from Thompson et al. (2018). The pipeline uses Trim Galore! 0.6.3 (https://www.bioinformatics.babraham.ac.uk/projects/trim_galore/ for QC), followed by the trimRRBSdiversityAdaptCustomers.py script from NuGen for trimming the diversity adapters. This script is available at https://github.com/nugentechnologies/NuMetRRBS.

All samples had comparable quality levels and no outstanding flags. Total number of reads was 45–90 million, with an average read length of ∼50 bp. Quality scores were mostly above 30 (including error bars), with the average above 38. Duplication level was reduced to <2 for ∼95% of the sequences.

High-quality reads were aligned to custom strain pseudogenomes, using Bowtie 2 (Langmead and Salzberg 2012) as implemented in Bismark 0.22 (Krueger and Andrews 2011). The pseudogenomes were created by incorporating strain-specific SNPs and indels into the reference genome using g2gtools, allowing a more precise characterization of methylation patterns. Bismark methylation extractor tool was then used for creating a BED file of estimated methylation proportions for each animal, which was then translated to the reference mouse genome (GRCm38) coordinates using g2gtools. Unlike other liftOver tools, g2gtools does not throw away alignments that land on indel regions. B6 samples were aligned directly to the reference mouse genome (mm10).

### Analysis of histone modifications

#### Identification of chromatin states

We used ChromHMM (1.22) (Ernst and Kellis 2017) to identify chromatin states, which are unique combinations of the four chromatin modifications; for example, one state could consist of high levels of both H3K4me3 and H3K4me1 and low levels of the other two modifications. We conducted all subsequent analyses at the level of the chromatin state.

Before running ChromHMM, we converted the BAM files that had been aligned to the B6 genome as described above to BED files using the BEDTools function bamtobed (Quinlan and Hall 2010). We then binarized the BED files using the BinarizeBed function in ChromHMM with default parameters.

We calculated chromatin states for all numbers of states between four and 16, which is the maximum number of states possible with four binary chromatin modifications (2^*n*^). We ran all mouse strains together in the same model as if they were different cell types in a standard run of ChromHMM.

To ensure we were analyzing the most biologically meaningful chromatin states, we aligned states across all models of four to 16 states by assigning each to one of the 16 possible binary states using an emissions probability of 0.3 as the threshold for presence/absence of the histone mark. This threshold was used for comparison purposes only and produced the most stable state estimates between models. We then investigated the stability of three features across all states: the emissions probabilities (Supplemental Fig. S8), the abundance of each state across transcribed genes (Supplemental Fig. S9), and the associations of each state with transcription (Supplemental Fig. S10). Methods for each of these analyses are described separately below. All measures were consistent across all models, but the 14-state model was characterized by a wide range of relatively abundant states with relatively strong associations with expression. We used this model for all subsequent analyses. For more details on how the different models were compared, see Supplemental Methods.

#### Genome distribution of chromatin states

We investigated genomic distributions of chromatin states using the ChromHMM function OverlapEnrichment to calculate enrichment of each state around known functional elements in the mouse genome. We analyzed the following features:

- Predicted liver chromatin states. We downloaded predicted liver chromatin states through the UCSC Genome Browser on February 14, 2023 (http://genome.ucsc.edu/cgi-bin/hgTables). We selected expression and regulation → chromatin state → cHMM liver P0 (encode3RenChromHmmLiverP0) under the mouse mm10 assembly. These data include chromatin state annotations for mouse liver on postnatal day 0. The annotations were based on ChIP-seq measurements of eight histone modifications: H3K27ac, H3K27me3, H3K4me3, H3K4me2, H3K4me1, H3K9me3, H3K9ac, and H3K36me3. ChromHMM was used to identify 15 chromatin states that were each annotated with a putative function based in the literature.
- CpG islands. Annotations of CpG islands in the mouse genome were included with the release of ChromHMM.
- Intergenic. Annotations of intergenic regions in the mouse genome were included with the release of ChromHMM.

### Gene body distribution of chromatin states

In addition to these enrichments around individual elements, we also calculated chromatin state abundance relative to the main anatomical features of a gene. For each transcribed gene, we normalized the base pair positions to the length of the gene such that the TSS was fixed at zero and the TES was fixed at one, taking into account the encoding strand of DNA. We also included 1000 bp upstream of the TSS and 1000 bp downstream from the TES, which were converted to values below zero and above one, respectively. To map chromatin states to the normalized positions, we binned the normalized positions into 42 bins running from −0.5 to 1.5. This range included some upstream and downstream regions around the gene body and gave us good resolution around zero and one. If a bin encompassed multiple positions in the gene, we assigned the mean value of the feature of interest to the bin. To avoid potential contamination from regulatory regions of nearby genes, we only included genes that were at least 2 kb from their nearest neighbor, for a final set of 14,048 genes.

#### Chromatin state and gene expression

We calculated the association of each chromatin state with gene expression (Fig. 2C). We did this both across genes and across strains. The across-gene analysis identified states that are associated with high expression and low expression within the hepatocytes. The across-strain analysis investigated whether variation in chromatin state across strains was associated with variation in gene expression across strains.

For each transcribed gene, we calculated the proportion of the gene body that was assigned to each chromatin state. We then fit a linear model separately for each state to calculate the association of state proportion with gene expression:

(1) $$y_{e}=\text{β}x_{s}+\epsilon ,$$

where *y*_*e*_ is the rank normal scores (Conover 1999) of the full transcriptome in a single inbred strain, and *x*_*s*_ is the rank normal proportion of each gene that was assigned to state*s*. We fit this model for each strain and each state to yield one β coefficient with a 95% confidence interval. We fit the strains independently to better identify variation in chromatin state effects across strains. However, the effects were not different across strains (ANOVA *P* > 0.5), so we averaged the effects and confidence intervals across strains to yield one summary effect for each state. We further fit models for each state independently, rather than using multiple regression, because we were primarily interested in the marginal effects of each state for this study.

To calculate the association of each chromatin state with gene expression across strains, we first standardized transcript abundance across strains for each transcript. We also standardized the proportion of each chromatin state for each gene across strains. We then fit the same linear model, where *y*_*e*_ was a rank normal vector concatenating all standardized expression levels across all strains, and *x*_*s*_ was a rank normal vector concatenating all standardized state proportions across all strains. We fit the model for each state independently, yielding a β coefficient and 95% confidence interval for each state.

In addition to calculating the association of state proportion across the full gene body with gene expression, we also performed the same calculations in a position-based manner (Fig. 5). To do this, we normalized the genomic positions of all chromatin states to run between zero at the TSS and one at the TES as described above. In dividing chromatin state values into bins, we averaged all positions for each state that were contained in each bin. We fit the linear model described above for each positional bin, thus creating position-based effect sizes for chromatin state on gene expression across genes and across strains.

### Analysis of DNA methylation

#### Creation of DNA methylome

We combined the DNA methylation data into a single methylome, cataloging all unique methylated sites across all strains. For each site, we averaged the percentage of methylation across the three replicates in each strain. The final methylome contained 5,311,670 unique CpG sites across the genomes of all nine strains. Because methylated CpG sites can be fully methylated, unmethylated, or hemi-methylated, we rounded the average percentage of methylation at each site to the nearest 0%, 50%, or 100%.

#### Decomposition of DNA methylome

To calculate the DNA methylation similarity across individuals shown in Figure 1B, we used the subset of the CpG sites that were shared across all strains at each B6 reference position. The resulting matrix contained individual mice in columns and shared methylation sites in rows. Each cell contained the measured level of DNA methylation at that position. We performed principal components analysis on this matrix.

#### Strain-specific CpG sites

In addition to the analysis of CpG sites that were shared across genes, we analyzed CpG sites that were strain specific. We defined a strain-specific CpG site as one that was present in all members of at least one strain and absent in all members of at least one other strain.

#### Distribution and methylation of CpG sites

We used the enrichment function in ChromHMM described above to identify enrichment of CpG sites around functional elements (e.g., CpG islands, mouse liver enhancers, and mouse liver promoters). These features are described above in the section “Genome distribution of chromatin states.” We further performed position-based analyses of both CpG density and percentage of methylation similar to the position-based abundance analyses performed for chromatin states.

To calculate overall CpG density relative to gene bodies, we calculated the inverse of the inter-CpG base pair distances within 1 kb of each expressed gene. We then normalized the position of each CpG to reflect its position relative to the gene's TSS (at zero) and its TES (at one) as described above. We took the average of these values in each of 42 bins, running from a relative position of −0.5 to 1.5. Figure 4C shows the average inverse inter-CpG distance across all 42 bins. CpG sites were most densely packed near the TSS (relative gene position = 0) as expected.

Figure 4D shows the average percentage of methylation in each of these bins, which was calculated in the same manner as above, but we calculated the median percentage of methylation in each bin rather than the inverse inter-CpG distance. The figure shows that CpG sites tended to be unmethylated near the TSS as expected.

#### Association of DNA methylation with gene expression

As with chromatin state, we assessed the association between DNA methylation and gene expression both across genes (Fig. 6A) and across strains (Fig. 6B). As with chromatin state, we binned the normalized CpG positions into 42 bins, running from just upstream of the TSS to just downstream from the TES. We treated missing CpG sites in individual strains as unmethylated, as it is uncommon for non-CpG sites to be methylated. This allowed us to test strain-specific CpG sites and variation in DNA methylation percentage simultaneously. We then fit the linear model shown in Equation 1, where *x*_*s*_ was the rank normal percentage of methylation either across genes or across strains in each position bin. Because the effect of DNA methylation on gene expression is well known to be dependent on position, we only calculated a position-dependent association with expression. We did not calculate the association of percentage of methylation across the full gene with expression.

#### Interactions between chromatin state and DNA methylation

We repeated the above analyses for DNA methylation conditioned on each of the 14 chromatin states. To do this, we isolated all CpG sites that were contained in the genomic regions defined by each chromatin state. We then performed the above analysis on each subset of CpG sites independently.

### Imputation of genomic features in DO mice

To assess the extent to which chromatin state and DNA methylation were associated with local expression QTLs, we imputed local chromatin state and DNA methylation into the population of DO mice. We compared the effects of the imputed epigenetic features to imputed SNPs and to local haplotype effects as measured in the DO.

All imputations followed the same basic procedure: For each transcript, we identified the haplotype probabilities in the DO mice at the genetic marker nearest the gene transcription start site. This matrix held DO individuals in rows and DO founder haplotypes in columns (Supplemental Fig. S11).

For each transcript, we also generated a three-dimensional array representing the genomic features (chromatin state, DNA methylation status, or SNP genotype) derived from the DO founders. This array held DO founders in rows, feature state in columns, and genomic position in the third dimension. The feature state for chromatin consisted of states one through 14; for SNPs, feature state consisted of the genotypes A, C, G, and T.

We then multiplied the haplotype probabilities by each genomic feature array to obtain the imputed genomic feature for each DO mouse. This final array held DO individuals in rows, the genomic feature in the second dimension, and genomic position in the third dimension (Supplemental Fig. S11). This array is analogous to the genoprob's object in R/qtl2 (Broman et al. 2019). The genomic position dimension included all positions from 1 kb upstream of the TSS to 1 kb downstream from the TES for the given transcript. SNP data for the DO founders in mm10 coordinates were downloaded from the Sanger SNP database (Keane et al. 2011) on July 6, 2021.

To calculate the association between each imputed genomic feature and gene expression in the DO population, we fit a linear model, $y_{e}=\text{β}x_{s}+\epsilon$, where *y*_*e*_ was DO gene expression of a single transcript, and *x*_*s*_ was the imputed level of a single chromatin state at a single-base-pair position within the encoding gene of the transcript. Before fitting this model, we regressed sex and DO generation out from all variables so that they would not be included in the estimate of variance explained by each chromatin state.

Testing each state separately is a bit artificial, because no single haplotype will explain as much variance as using all haplotypes together in a multiple regression. However, it was critical in this study to maintain a single degree of freedom across all features so that we could compare them. Otherwise, haplotypes have seven degrees of freedom (df) at each location; chromatin states potentially have 13 df, although in practice they typically have between two and four df; and both SNPs and DNA methylation have only one df. Thus, to compare the features, we tested only a single state at a time. From these linear models, we calculated the variance explained (*R*^2^) by each genomic feature at each position (Fig. 7), thereby relating gene expression in the DO to each position of the imputed feature in and around the gene body. We also kept the β coefficients to identify overall trends in positive or negative associations on gene expression for each genomic feature at each position (Fig. 5C).

#### Permutations

Because any feature imputed from haplotype will be correlated with any feature that haplotype is correlated with, we performed permutations of the above statistics to assess whether each genomic feature was significantly correlated with gene expression beyond the effect of the imputation itself. To do the permutations, we shuffled the strain labels on each genomic feature vector (chromatin states, DNA methylation percentage, or SNPs). This randomized the association between haplotype and the assigned genomic feature while preserving the association between haplotype and gene expression. We then reimputed the permuted features into the DO and performed the association tests on the randomized imputed values as described above.

We performed 1000 permutations for each transcript, retaining the *R*^2^ value from each permutation. We then calculated an empirical *P*-value for the *R*^2^ of each transcript based on these permutations. This was the number of times the permutations met or exceeded the observed *R*^2^ value divided by the total number of permutations. We then analyzed the empirical *P*-value distributions for uniformity. A uniform *P*-value distribution across the transcripts would suggest that the given genomic feature was not significantly associated with gene expression. An enrichment of small *P*-values, on the other hand, would suggest that there is a significant association between the imputed genomic feature and gene expression beyond that conferred by the imputation itself. The *P*-value distributions for all three genomic features were highly enriched for small *P*-values (all Kruskal–Wallis *P* < 2^−16^), suggesting that, although many individual imputed values were not significantly associated with gene expression, overall each genomic feature could be significantly associated with gene expression (Supplemental Fig. S6).

## Data access

All raw and processed sequencing data generated in this study have been submitted to the NCBI Gene Expression Omnibus (GEO; https://www.ncbi.nlm.nih.gov/geo/) under accession number GSE213968. Code to run the analyses in this study is available as Supplemental Code and at GitHub (https://github.com/annaLtyler/Epigenetics_Manuscript).

## Supplementary Material

Supplement 1

Supplement 2
