# Supplementary material for: Variation in histone configurations correlates with gene expression across nine inbred strains of mice
Source: Genome Res. 2023 Jun;33(6):857–71. doi: 10.1101/gr.277467.122 (PMC10519406; doi:10.1101/gr.277467.122)
Supplement: Supplement 2 [file Supplemental_Figures.pdf]

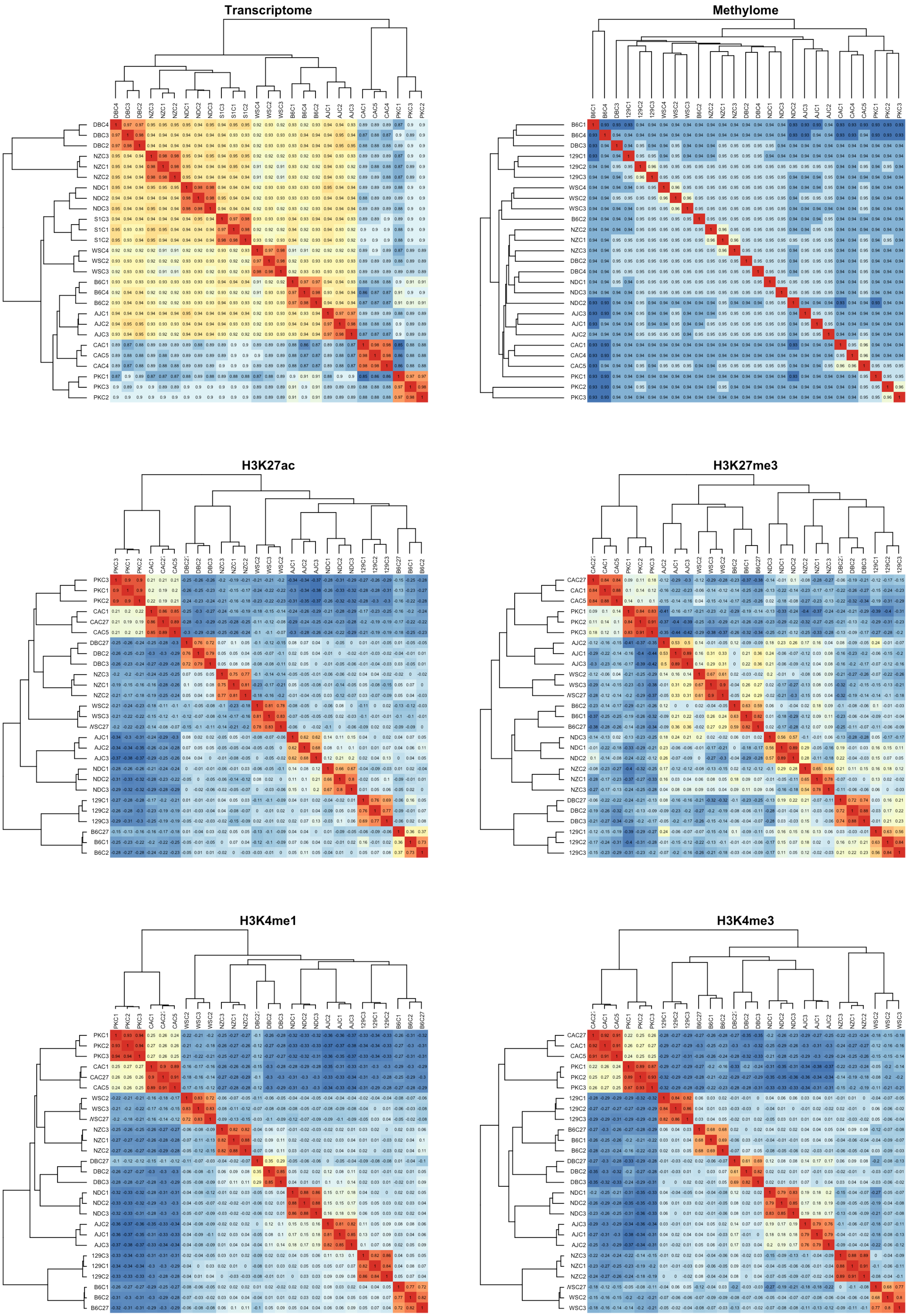

Supp. Fig. S1

Correlations of multiple genomic features across inbred mice. Each panel shows one genomic feature A. Transcriptome; B. Methylome; C. H3K27ac; D. H3K27me3; E. H3K4me1; F. H3K4me3. These figures complement Figure 1 in the manuscript and show a more finely detailed structure of the correlations between individuals and strains.

Supp. Fig. S2

	Intergenic	CpG Island	Poised Enh. (Tsd)	Poised Enh. (Tsp)	Strong Enh. (Tsd)	Strong Enh. (Tsp)	Weak Enhancer	Hetero. (polycomb)	Active Promoter	Bivalent Promoter	Promoter (FR)	Weak Promoter	Transcription Initiation
State 1	2.11	7.11	8.2	14.6	20.6	29.8	17.6	0.76	19.4	6.91	30.1	20.9	17.2
State 2	2.43	0.21	10.5	1.5	9.32	2.89	8.83	0.39	0.14	0.38	0.54	0.28	4.21
State 3	0.36	132	1.09	7.57	2.03	11.4	2.57	0.49	134	11.2	5.89	48.8	2.74
State 4	2.91	0.41	3.6	1.35	3.4	1.93	3.47	0.57	0.26	0.26	0.4	0.38	1.84
State 5	0.09	44.8	1.17	15.5	1.43	13.5	6.04	3.59	29.8	75.1	14.1	43.4	2.46
State 6	2.16	0.21	5.67	2.54	1.58	0.76	1.44	0.48	0.07	0.47	0.56	0.41	2.86
State 7	0.06	22	2	9.47	5.06	12.2	7.19	8.94	6.27	46.3	5.36	9.61	3.95
State 8	0.45	3.85	7.35	27.7	1.37	4.77	0.98	1.78	3.55	9.16	40	26.7	37.2
State 9	0.5	1.81	2.58	9.35	1.22	3.23	1.17	2.82	1.32	1.96	5.02	6.95	8.3
State 10	0.22	24.8	1.19	11.4	0.68	1.46	1.03	16	1.08	82.4	5.55	7.33	2.38
State 11	0.05	16	0.61	19.3	0.46	5.2	0.54	24	8.89	66.5	13.4	33.7	3.26
State 12	0.07	57.2	0.26	22	0.13	4.13	0.27	7.62	9.77	197	31.8	56.1	2.86
State 13	14.1	0.84	0.2	0.65	0.14	0.17	0.22	5.26	0.08	1.88	0.13	0.24	0.2
State 14	74.5	0.07	0.35	0.27	0.22	0.13	0.3	0.16	0.02	0.03	0.04	0.07	0.22

This figure corresponds to Figure 2B, which shows enrichment of each state around predicted functional elements in the mouse genome. Instead of scaled values, this figure shows raw fold enrichment values with the enrichment value printed in each cell.

Supp. Fig. S3

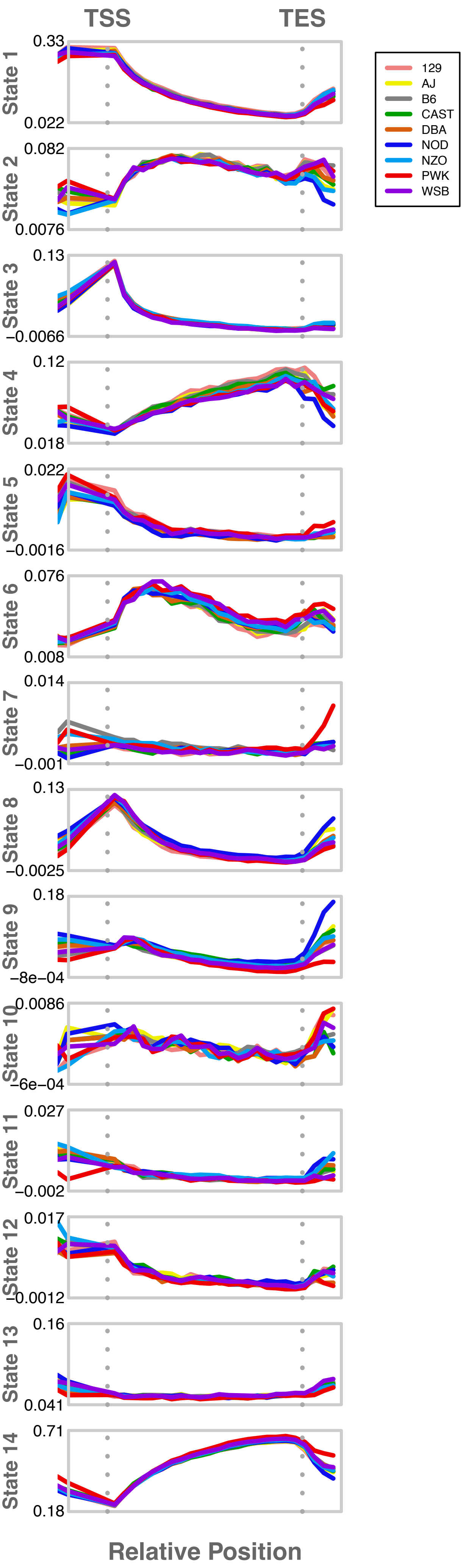

These figures are companions to those shown in Figure 4A. Each panel shows the abundance of a single state for each strain. The color of each line indicates the strain. The abundance pattern of each state is largely identical across the strains. Deviations are seen only outside the gene body for very rare states are are thus artifacts due to low numbers of example genes.

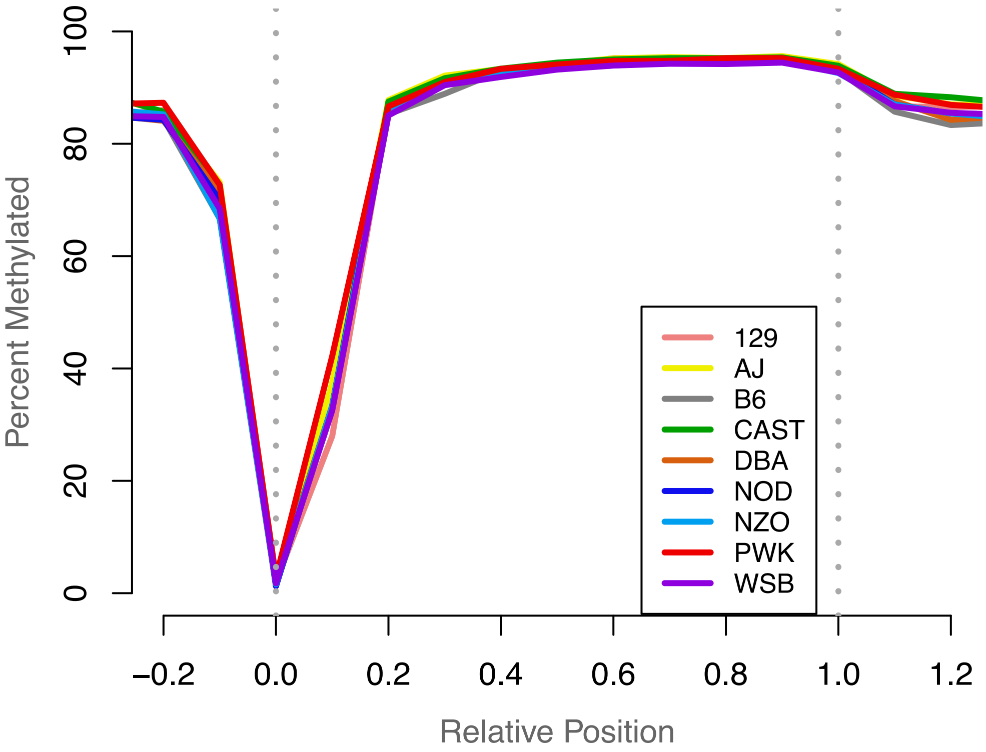

Supp. Fig. S4

This figure is a companion to the DNA methylation percent panel in Figure 4I It shows DNA methylation percent for strain along the gene body. Strain is indicated by color.

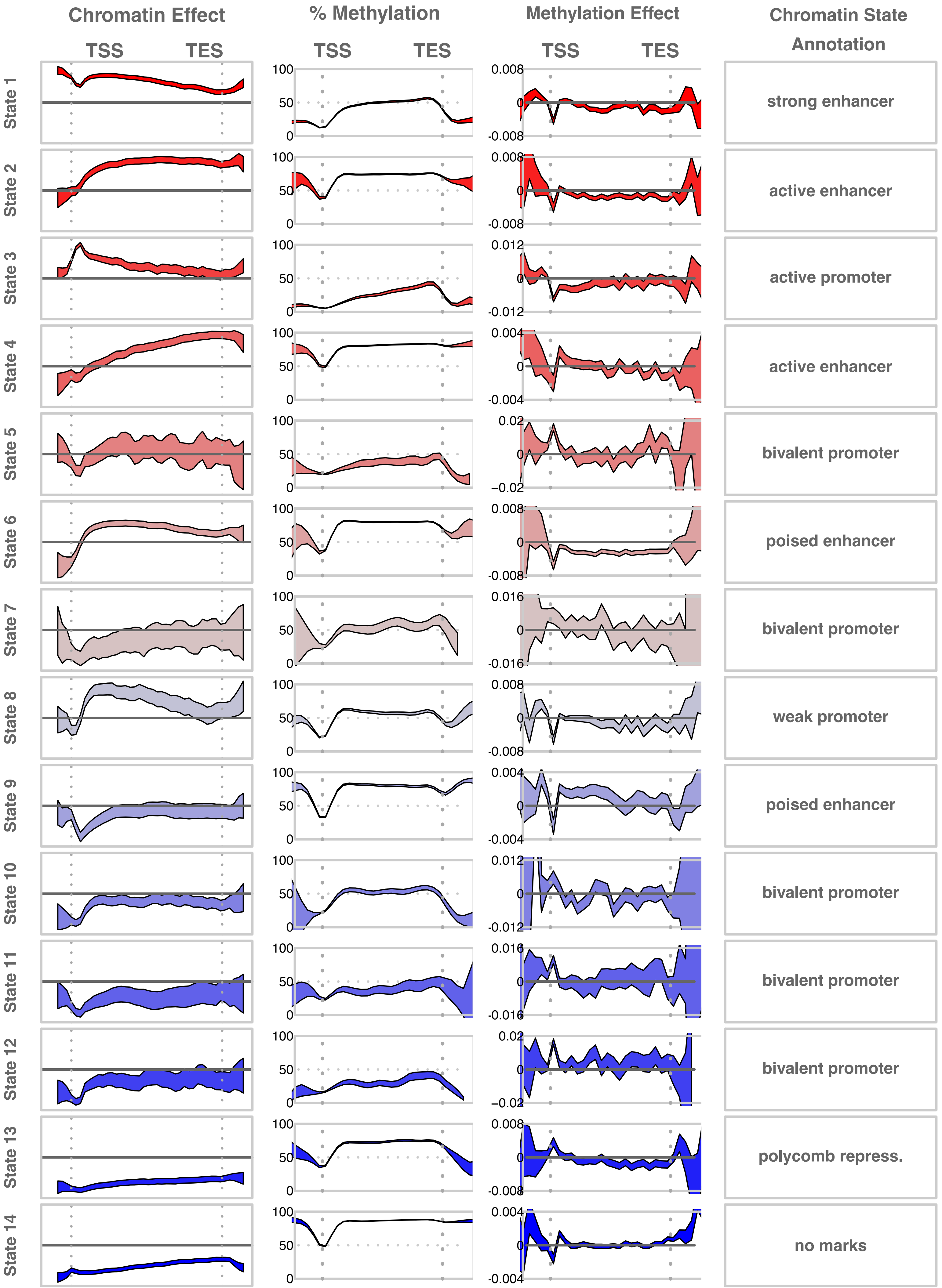

Supp. Fig. S5

Comparison of chromatin state effects on gene expression to the effects of DNA methylation in each chromatin state on gene expression. Columnn 1: The association of each chromatin state to gene expression. Column 2: The percent DNA methylation of CpG sites in each state. Column 3: The association between DNA methylation in each state with gene expression. Column 4: The state annotation for reference. Note that DNA methylation in each state tends to be associated with expression in a manner opposite to that of the chromatin state. If the chromatin state had a positive association with gene expression, DNA methylation in that state had a negative association with gene expression and vice versa.

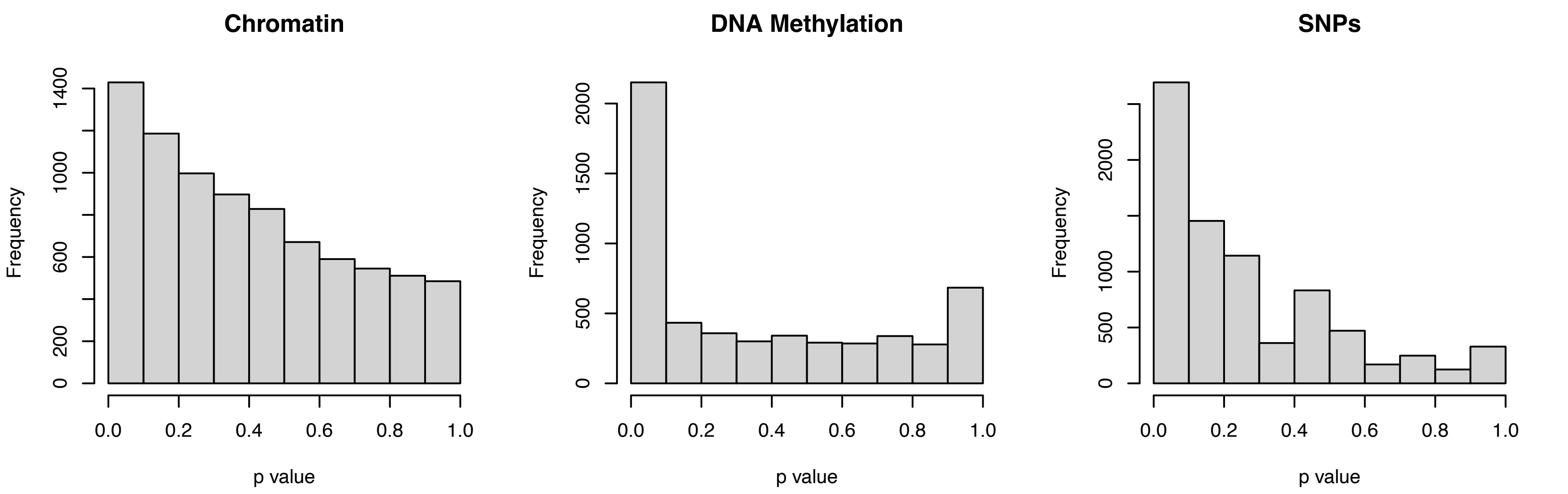

Supp. Fig.6

These figures show empirical p–value distributions obtained by permuting imputed genomic features and associating them with gene expression in the DO mice. All distributions are enriched with small values suggesting that overall each genomic feature is associated with gene expression in the DO beyond the simple correlation with haplotype.

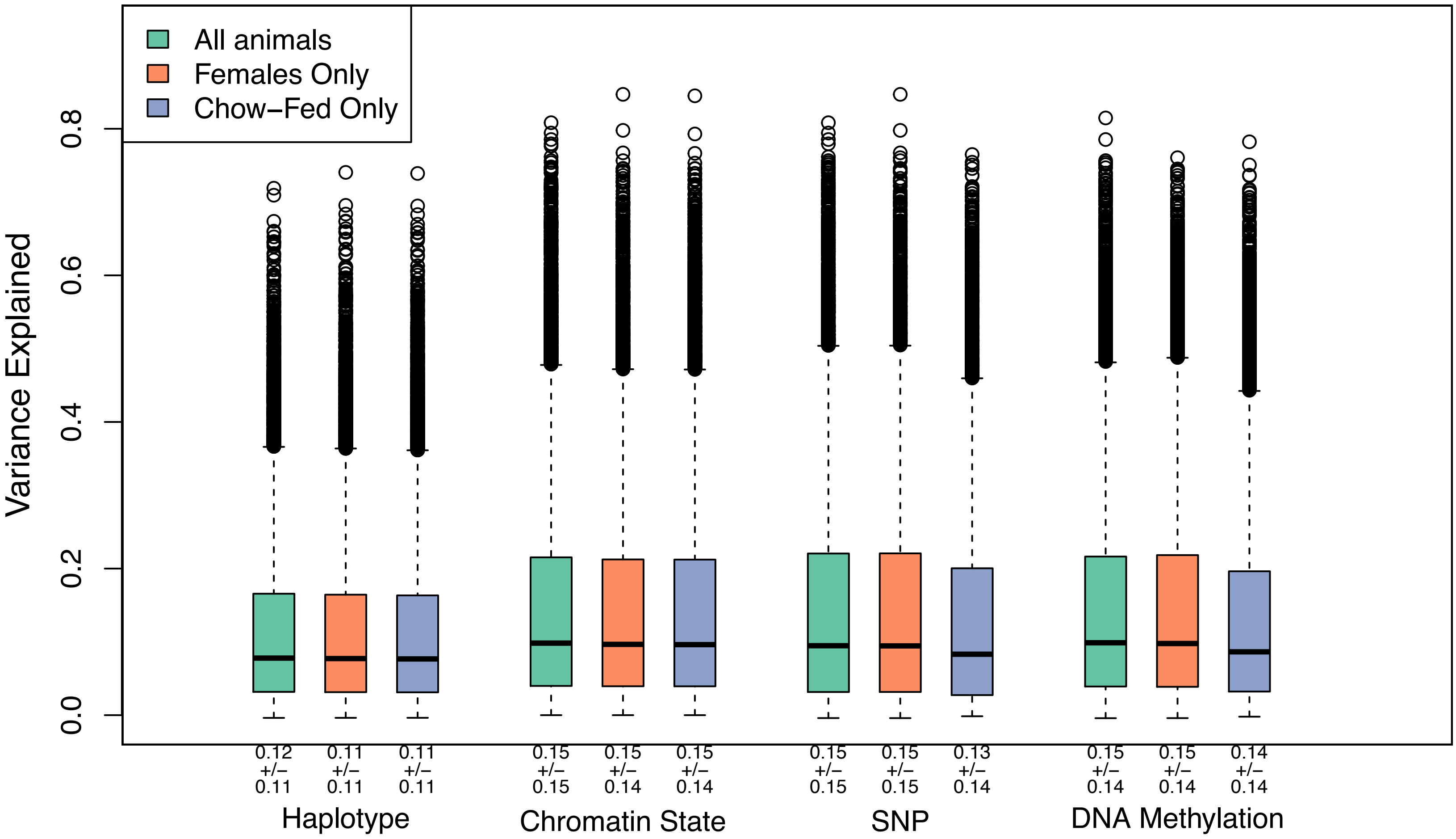

Supp. Fig.7

Each box shows the distribution of variance explained by each genomic feature for all transcripts in the DO liver. These distributions are comparable to those in Fig. 7. Boxes are grouped by genomic feature. They are colored by which subset was being tested: all animals (green), just females (orange), just chow−fed animals (purple). Values below each box indicate the mean value (top) and standard deviation (bottom) for each distribution. For each genomic feature the different subsets of the data yield nearly identical results.

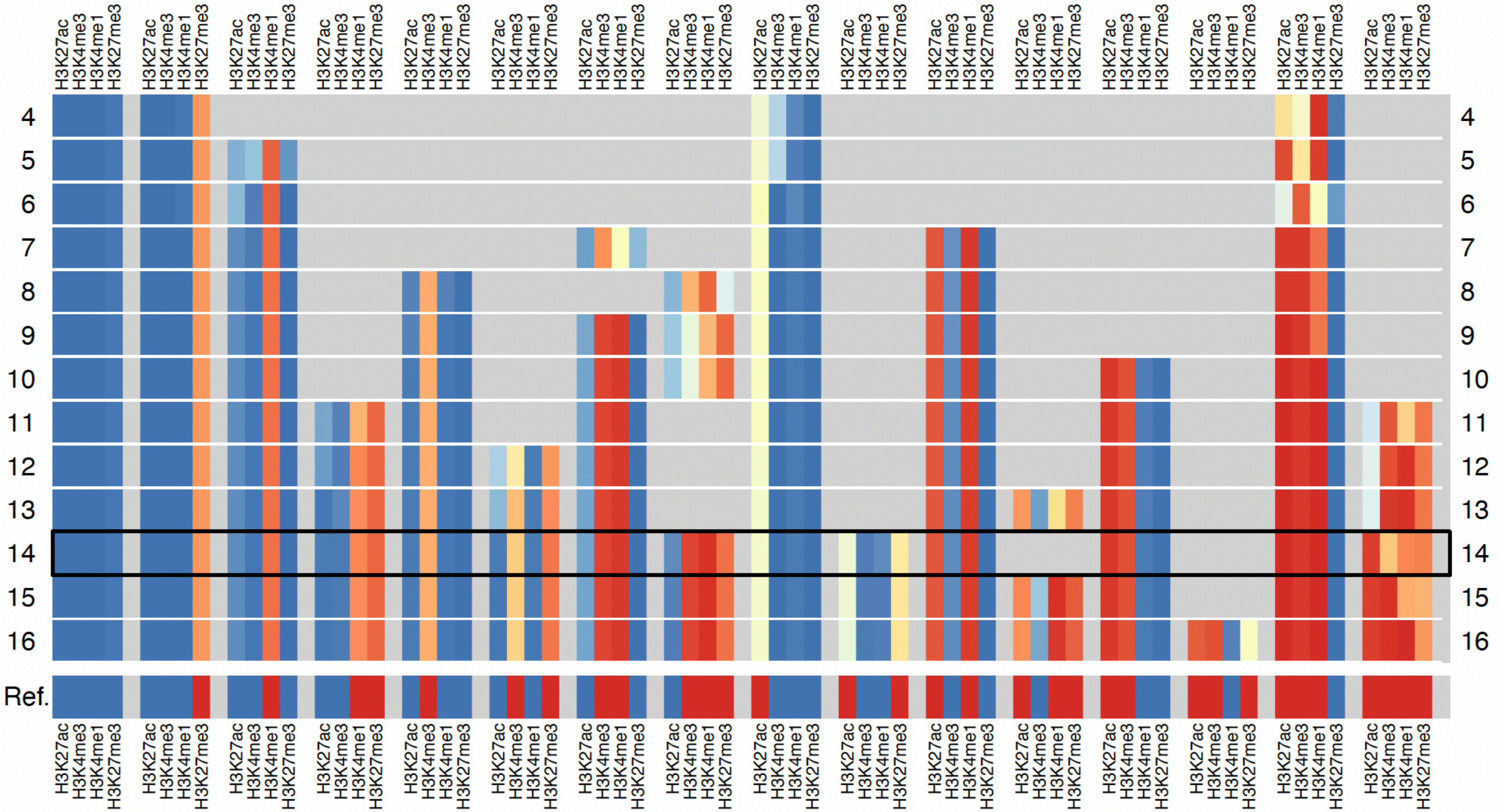

Supp. Fig. 8

Comparison of emissions probabilities for each histone mark in each model ranging from 4 states to 16 states. Red indicates a probability of 1 and blue indicates a probability of 0. Each histone mark is indicated along the top and bottom of the figure. The bottom row shows the binary state values for reference. The black outline highlights the model that was used in this study: the 14-state model. Overall, states were stable in their emissions spectrum across the models.

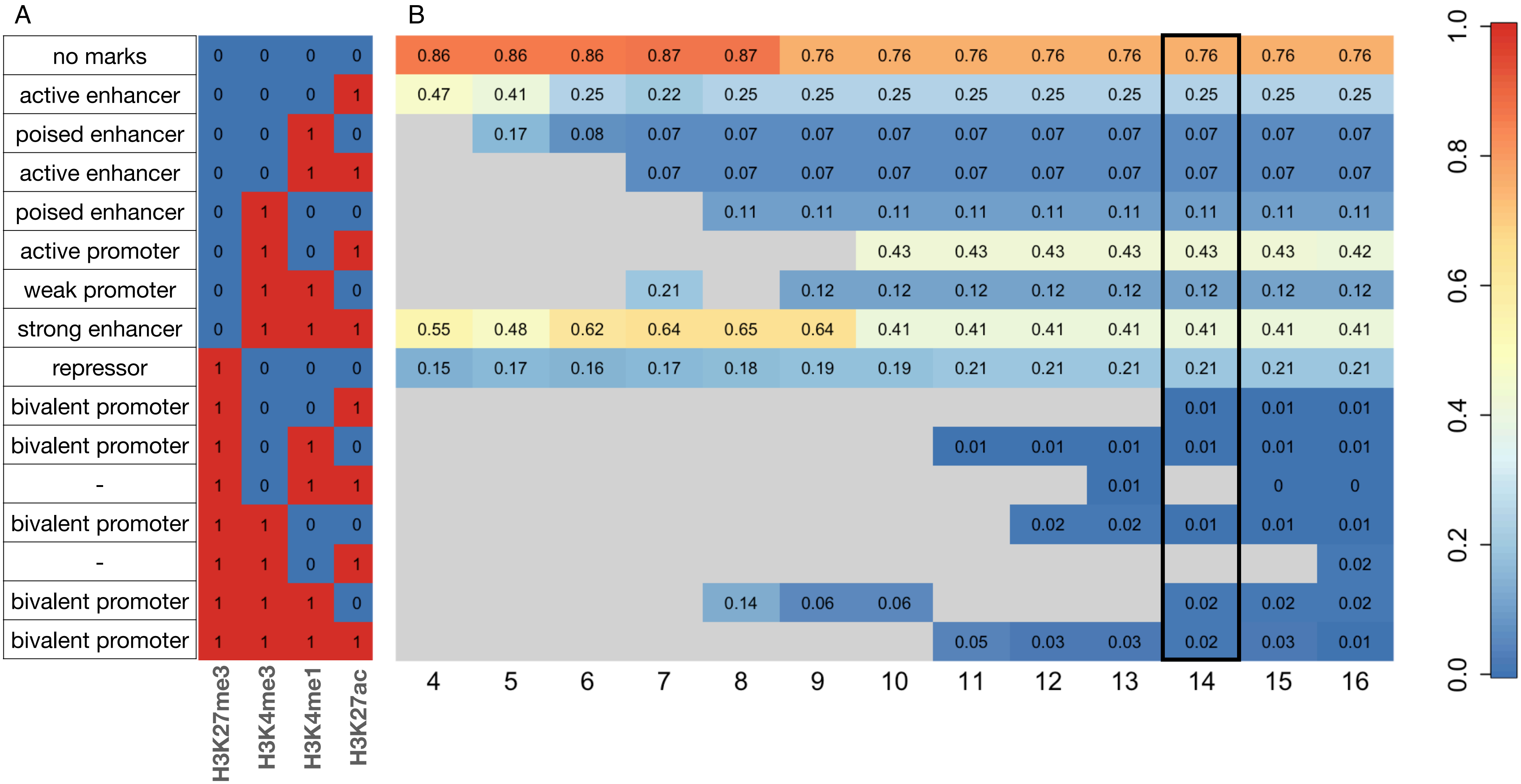

Supp. Fig. 9

Comparison of the abundance of each state across ChromHMM models ranging from four states to 16 states. A. Annotations and idealized emissions for each state. B. Each column shows the results for a different ChromHMM model. Each cell shows the beta coefficient from a linear model testing the association between state abundance and gene expression in the inbred mice. Each cell is colored based on the sign and magnitude of the beta coefficient for ease of visualization. States that were not present in a given model are shown in gray. The black box outlines the 14-state model, which was used in this paper. Overall, the state assignments were stable across models and had consistent effects on gene expression.

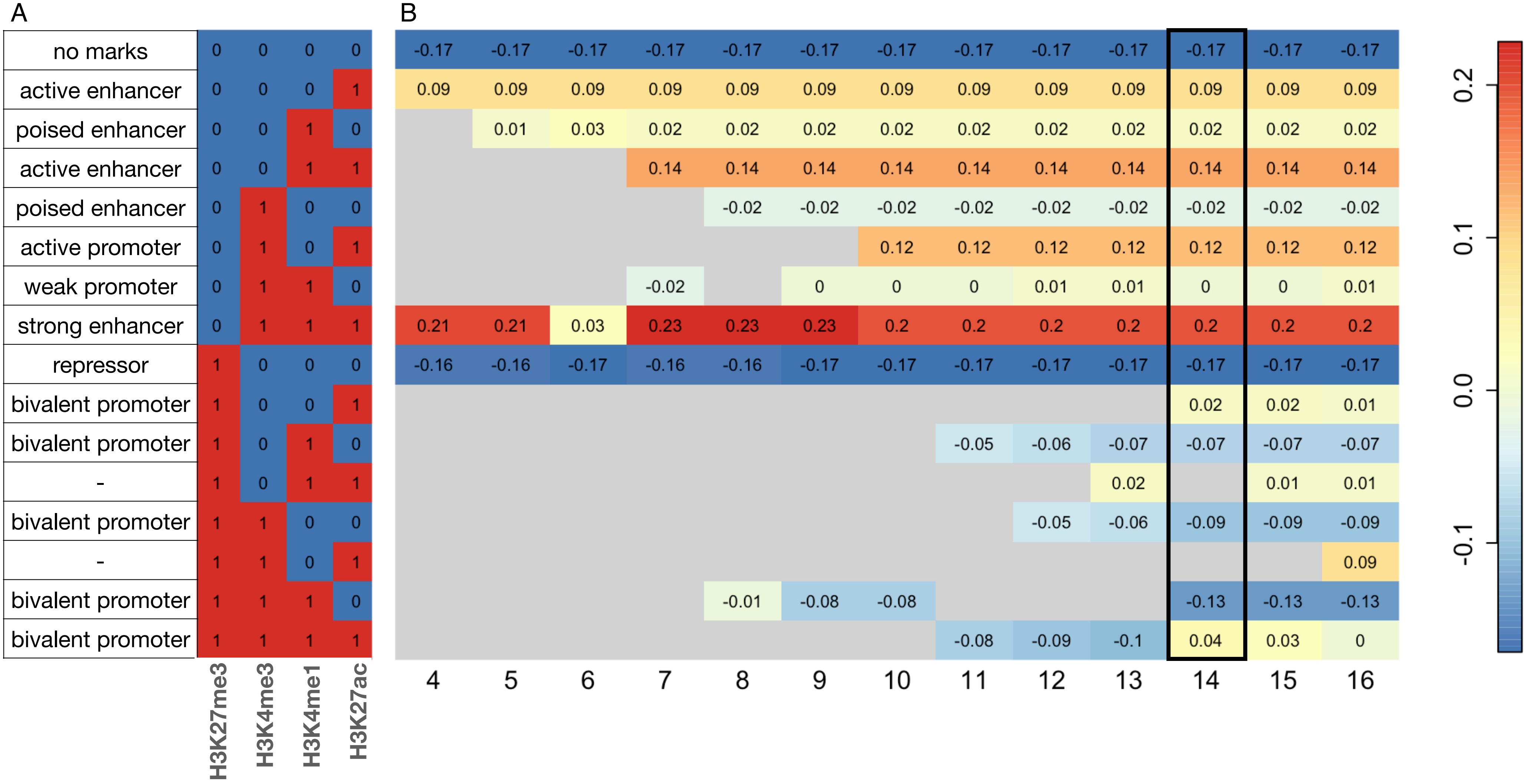

Supp. Fig. 10

Comparison of the correlation between state abundance and gene expression in the inbred mice across ChromHMM models ranging from four states to 16 states. A. Annotations and idealized emissions for each state. B. Each column shows the results for a different ChromHMM model. Each cell shows the beta coefficient from a linear model testing the association between state abundance and gene expression in the inbred mice. Each cell is colored based on the sign and magnitude of the beta coefficient for ease of visualization. States that were not present in a given model are shown in gray. The black box outlines the 14-state model, which was used in this paper. Overall, the state assignments were stable across models and had consistent effects on gene expression.

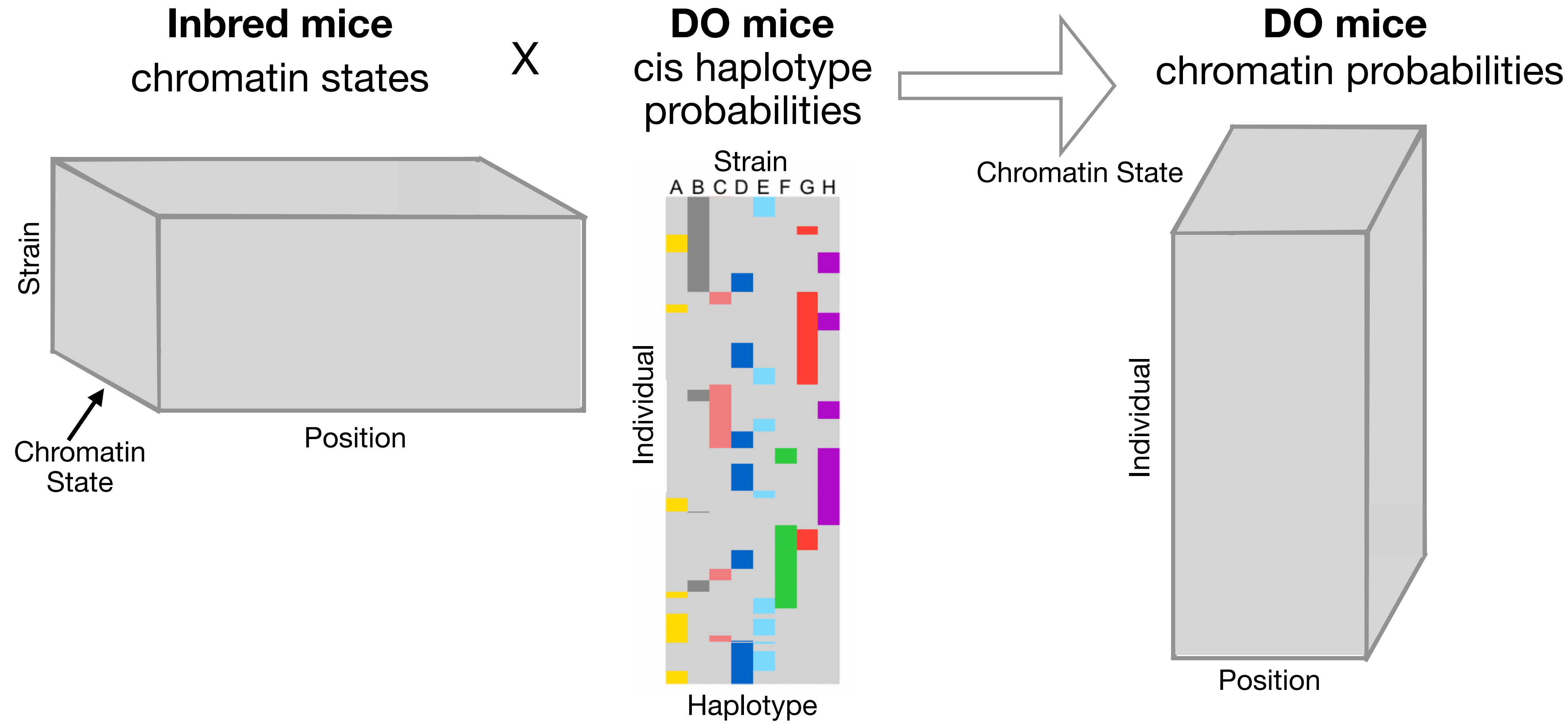

Supp. Fig. 11

The general schematic for imputing chromatin state and other genomic features into the Diversity Outbred (DO) mice. A genomic feature matrix with three dimensions (position x feature x strain) was multiplied by the haplotype probability matrix for the marker nearest the gene of interest. The result was a three-dimensional matrix (position x DO individual x imputed feature probability).
